# SPOmiAlign: a modality-agnostic computational framework for multimodal spatial omics alignment enabled by a feature matching foundation model

**DOI:** 10.1093/bib/bbag331

**Published:** 2026-06-21

**Authors:** Yi Wang, Zihang He, Yunjie Yan

**Affiliations:** Liangzhu Laboratory, Zhejiang University School of Medicine, No. 1369 Wenyi West Road, Yuhang District, Hangzhou, Zhejiang Province 311113, China; Zhejiang Key Laboratory of Multi-Omics in Infection and Immunity, Center for Infectious Disease Research, School of Medicine, Westlake University, No. 18 Shilongshan Street, Zhuantang Subdistrict, Xihu District, Hangzhou, Zhejiang Province 310024, China; Zhejiang University-University of Edinburgh Institute (ZJE), Zhejiang University School of Medicine, Zhejiang University, 718 East Haizhou Road, Haining, 314400, China; Sir Run Run Shaw Hospital, School of Medicine, Zhejiang University, 3 East Qingchun Road, Hangzhou, 310016, China

**Keywords:** spatial multimodal alignment, feature matching, foundation model, common coordinate framework, spatial multi-omics integration

## Abstract

Multimodal spatial omics enables systematic characterization of tissue organization by jointly profiling transcriptomic, proteomic, metabolomic, and other spatially resolved modalities within their spatial context. A central challenge in realizing this potential is achieving robust spatial alignment across modalities and sections. Although numerous alignment methods have been developed, most are designed for single-modality sections or specific modality combinations, with few enabling modality-agnostic alignment. Cross-modal alignment remains difficult due to the absence of shared molecular features, partial spatial overlap, and nonrigid tissue deformations. To address these challenges, we introduce SPOmiAlign, a modality-agnostic framework for spatial multimodal alignment, enabled by a feature-matching foundation model that serves as a general computational primitive for spatial multi-omics alignment. The framework enables accurate cross-modal spatial alignment without manual intervention or modality-specific tuning. Across diverse multimodal benchmarks, SPOmiAlign consistently achieves higher alignment accuracy than existing methods. We further demonstrate its utility through automated registration to a common coordinate framework, enabling standardized anatomical annotation. Finally, applying SPOmiAlign to integrate spatial transcriptomic, proteomic, and metabolomic data in mouse brain facilitates the identification of spatial domains that were difficult to resolve with less accurate registration, highlighting its utility for multi-omic integration and biological interpretation.

## Introduction

Recent advances in spatial transcriptomics (ST), proteomics, and metabolomics have expanded the landscape of spatial multi-omics, enabling *in situ* measurement of diverse biomolecular modalities within tissue sections [1]. As complementary spatial modalities are increasingly profiled in adjacent or parallel sections, establishing an accurate correspondence between them becomes essential [2–4]. Precise cross-modal alignment of tissue sections is therefore a fundamental prerequisite for integrative spatial analysis.

In recent years, numerous computational methods have been proposed for spatial tissue section registration. Existing approaches broadly fall into three categories: image-to-image registration [5–8], single-modality spatial omics alignment, which was initially dominated by ST approaches [9–19], and more recently, spatial multiomic alignment frameworks [20, 21] emerging alongside multimodal experimental protocols. In parallel, tools have been developed for spatial omic-to-image alignment, integrating molecular measurements into histological references [22, 23].

Despite these advances, existing approaches for cross-modal alignment remain limited. First, most cross-modal spatial alignment methods are designed for two specific modalities [21, 22], restricting their applicability to the simultaneous alignment of spatial transcriptomic, proteomic, and metabolomic sections. In addition, cross-modal alignment is often complicated by severe geometric deformations and nonoverlapping regions. Although methods such as ELD [20] demonstrate cross-modal capability, performance can degrade under complex spatial distortions, including partial tissue overlap, limiting robustness in practical scenarios. Therefore, these challenges underscore the need for a generalizable and robust cross-modal alignment framework to enable integrative multimodal spatial analysis.

To overcome the limitations of existing spatial alignment tools for spatial multi-omics data, we developed SPOmiAlign as a modality-agnostic alignment framework for heterogeneous tissue sections. Its key methodological advances are as follows: (i) Unified representation: SPOmiAlign transforms diverse spatial omics data into a unified spatial structural image (SSI) representation, allowing alignment across transcriptomic, proteomic, metabolomic, and histological modalities. (ii) Dense correspondence-based alignment: it reformulates cross-modal alignment as a dense correspondence problem rather than relying primarily on iterative optimization or manually defined landmarks. (iii) Edge-enhanced matching: it introduces an edge-enhanced matching strategy that strengthens boundary-aware correspondences, which are especially important for tissue alignment but are often underrepresented in generic feature matching. (iv) Spot-level reassignment: it incorporates a reassignment module to recover one-to-one spot correspondence after warping across modalities with different resolutions and spot densities. (v) Downstream-oriented design: SPOmiAlign is designed not only for accurate registration, but also for downstream applications such as atlas mapping and integrative spatial multi-omics analysis.

To support this dense-correspondence-based design, we further adopted a feature matching backbone that is particularly suitable for heterogeneous tissue alignment. Existing feature matching methods include sparse matching pipelines such as SuperPoint [24] and LightGlue [25] and detector-free or dense matching models such as LoFTR [26] and robust dense feature matching (RoMa) [27]. Among them, we chose RoMa as the backbone of SPOmiAlign for three main reasons. First, as a dense matcher, RoMa provides substantially richer and more spatially distributed correspondences than sparse keypoint-based methods, which is particularly advantageous for tissue sections with continuous structures, complex boundaries, and weak local textures. Second, by combining global features with fine features, RoMa can capture both large-scale tissue organization and fine-grained local structures, making it well suited to spatial multi-omics images that contain information at multiple scales. In addition, RoMa outputs not only dense correspondences, but also reliable matching certainties, offering better interpretability and providing a principled basis for our subsequent edge-aware reweighting strategy to emphasize biologically informative tissue boundaries.

Extensive benchmarking demonstrates that SPOmiAlign achieves superior registration accuracy while significantly reducing computational overhead. In particular, our framework enables the automated registration of spatial omics data to a common coordinate framework (CCF) for standardized anatomical annotation. Furthermore, by applying SPOmiAlign to a mouse brain spatial multiomic dataset, including ST, spatial proteomics (SP), and spatial metabolomics (SM), we demonstrate that the accurate alignment achieved by SPOmiAlign facilitates spatial domain refinement and integrative spatial multiomic analysis.

## Methods

The article should have specific wording and appear in a specific order

### Data preprocessing in SPOmiAlign

**Generation of SSIs.** To convert spot-based spatial omics matrices into an image-form representation suitable for dense correspondence matching, we rasterize spatial coordinates and molecular intensities into a single-channel *SSI*. SSIs preserve tissue morphology at meso-scale resolution and serve as structural input for downstream alignment within SPOmiAlign.

Given a spatial omics dataset with $N$ spots, each spot $i$ is represented by a spatial coordinate $(u_{i}, v_{i})$ and a vector of molecular features $D$ of dimensions $\mathbf{x}_{i} \in \mathbb{R}^{D}$. The spot intensity is aggregated into a scalar value:


\begin{align*} & I_{i} = f(\mathbf{x}_{i}). \end{align*}

By default, we adopt feature summation to produce a unified structural signal (e.g. total UMI counts for ST).


\begin{align*} & I_{i} = \sum_{d=1}^{D} x_{id}. \end{align*}

Alternatively, scalar intensities can also be extracted directly from metadata fields (obs), allowing flexibility for transcriptomic, proteomic, and metabolomic datasets.

The raw intensity values optionally undergo the transformation $\log (1+I_{i})$, followed by clipping to the first and 99th percentiles to suppress extreme outliers and min-max normalization to $[0,1]$. A user-defined percentile threshold may be applied to retain high-confidence spots if desired.


\begin{align*} & \hat{I}_{i} = \frac{\operatorname{clip}(I_{i}^{\prime}) - P_{1}}{P_{99}-P_{1}}, \qquad \text{and optionally}\ \hat{I}_{i}> t. \end{align*}

Spatial coordinates are automatically mapped to a 2D canvas while preserving aspect ratio. Coordinates are shifted to nonnegative space and discretized into pixel indices. Each spot is rendered onto the canvas using circular or square kernels of a specified radius, producing a single-channel grayscale image.


\begin{align*} & \mathrm{SSI}(x,y) = \operatorname{Rasterize}(u_{i},v_{i},\hat{I}_{i}). \end{align*}

High-intensity spots are drawn with stronger pixel responses (white-on-black or black-on-white depending on configuration). When regions overlap, compositing is performed using min/max blending to preserve relative spot density. The final output is saved as an 8-bit grayscale PNG without interpolation between spots.

In summary, SSIs provide a unified structural representation of spatial omics data by aggregating molecular features and rasterizing spatial spot distributions into dense grayscale images, enabling efficient image-based alignment in SPOmiAlign. Alternatively, high-dimensional omics profiles can be projected into low-dimensional spaces via principal components or cell-type annotations and encoded as RGB images to preserve richer spot information.

**Registering Slide-seq mouse brain coronal sections to the Allen Brain Atlas.** We used the Slide-seq mouse brain ST dataset comprising 101 coronal sections spanning the anteroposterior axis of the adult mouse brain. Following the original study, we selected two representative sections (sections 29 and 43) for registration in the Allen Brain Atlas. For each selected section, we constructed the corresponding SSI as described above. To enhance anatomical contrast in SSIs, spatial spots are filtered by RNA abundance and retained only spots whose total UMI counts were within the top 20% of the section (i.e. above the 80th percentile of the total UMI counts distribution). The original Slide-seq data have a pixel resolution of 1.3 $\mu$m. During the SSI rendering, each retained spot was drawn as a filled disk with a radius of 5 pixels, corresponding to an effective spatial coverage of $\sim$13 $\mu$m per spot.

Each Slide-seq section was accompanied by a paired Nissl-stained histological image. For section 29, the SSI exhibited sufficient anatomical contrast to allow direct alignment to the corresponding Nissl reference image of the Allen Brain Atlas. Accordingly, the SSI of section 29 was directly registered to the atlas Nissl image to obtain CCF coordinates. In contrast, for section 43, direct registration of the SSI to the atlas Nissl image resulted in suboptimal alignment performance due to reduced boundary structural contrast in the SSI. To address this, we adopted a two-stage alignment strategy for section 43. Specifically, the SSI was first aligned to its paired Nissl-stained image, leveraging the high structural correspondence between the ST section and its histological reference. Subsequently, the paired Nissl image was mapped to the corresponding Nissl reference image of the Allen Brain Atlas. The composed transformation from these two steps was then applied to map the SSI of section 43 into the CCF.

**Registering MERFISH sagittal mouse brain sections to the Allen Brain Atlas.** To evaluate the applicability of SPOmiAlign to mouse brain sagittal sections and imaging-based ST, we registered a representative MERFISH sagittal section (midline section 008 among 25 sagittal sections) to the Allen Brain Atlas. We first constructed the SSI for the selected MERFISH section. During SSI rendering, each spot was rendered as a circular spot with a radius of 1 pixel.

**Anatomical annotation from the Allen Brain Atlas.** The Allen Brain Atlas provides high-resolution anatomical reference images together with a systematic and hierarchical taxonomy of mouse brain structures. In this study, we used the coronal atlas (132 coronal sections spaced at 100 $\mu$m intervals) and the sagittal atlas (21 sagittal sections spaced at 200 $\mu$m intervals). Each atlas includes Nissl-stained images and the corresponding anatomical annotations. For annotation retrieval, the required inputs include the index of the CCF reference section corresponding to the experimental slice and an anatomical annotation volume. Specifically, Slide-seq coronal sections 29 and 43 were mapped to CCF coronal sections 205 and 273, respectively, while MERFISH sagittal section 008 was mapped to CCF sagittal section 130 by manual inspection. Anatomical annotations were obtained from the Allen CCF 2017 release, which provides annotation volumes at multiple isotropic resolutions (10, 25, 50, and 100 $\mu$m). We used the annotation_25.nrrd volume, which stores an integer brain-region identifier for each voxel coordinate $(x,y,z)$ in the CCF. For a given CCF reference section, a 2D annotation map was obtained by extracting the plane corresponding to the matched index (e.g. $z=205$) from the annotation volume. Different colors were assigned to different region identifiers to generate a 2D annotation image for visualization and downstream processing. Because the annotation volume (25 $\mu$m isotropic resolution) differs from the pixel resolution of the Nissl-stained CCF images, a coordinate mapping between the Nissl-stained image and the annotation map is required. The regions of interest (ROIs) were independently extracted from the Nissl-stained image and the corresponding annotation image using Grounding DINO [28]. The Nissl ROI was resized to match the annotation ROI and then padded back to the original annotation image size, yielding a coordinate transformation between the two reference spaces. Anatomical annotation retrieval was performed in two steps. First, the experimental section was aligned to the corresponding Nissl-stained image. Second, the same coordinate transformation was applied to map the aligned experimental coordinates to the annotation space, after which anatomical annotations were assigned to the experimental spots.

**Spatial Multiomic dataset of the mouse brain (MISAR-seq).** We used a spatial multiomic dataset of the MISAR-seq containing paired ST and ATAC-seq spatial sections of two samples. To increase the cross-modality discrepancy for validation, we introduced an affine transformation to the Sample 2 section, generating a simulated Sample 2 slice. The ST section (Sample 1) was used as a reference, while the spatial ATAC-seq section (Sample 2) served as a source. Each section was associated with a corresponding H&E-stained image. We first aligned the two H&E images to estimate a warping transformation. The resulting transformation was then applied to the spatial coordinates of all the spots in the source section to achieve multiomic spatial alignment.

**cRCC ST and metabolomics dataset.** We evaluated SPOmiAlign on a clear cell renal cell carcinoma (cRCC) spatial multi-omics dataset consisting of ST and MALDI imaging mass spectrometry-based metabolomics. To enable cross-modal alignment, we constructed SSIs for the two modalities and aligned the SM data to the ST reference space. For the spatial transcriptomic SSI, spot intensities were defined using total UMI counts. For the spatial metabolomic SSI, spot intensities were derived from measured metabolite intensities. In SSI construction, each spatial location was rendered as a square spot, with spot size determined according to the spatial resolution and visual representation requirements of each modality. All SSIs were constructed and visualized in Python using Matplotlib.

**Mouse brain spatial tri-omics dataset.** We further evaluated SPOmiAlign on a spatial tri-omics mouse brain dataset integrating MAGIC-seq-based ST, MALDI imaging mass spectrometry-based metabolomics, and PLATO based SP. We first performed manual rotation and scaling between sections to obtain a reference alignment and then constructed SSIs for downstream comparison. For spatial transcriptomic SSIs, spot intensities were defined using total UMI counts. For spatial metabolomic and proteomic SSIs, spot intensities were derived from measured metabolite and protein intensities, respectively. In the spatial transcriptomic and proteomic SSI, each spot was rendered as a square spot of radius 15, while in the spatial metabolomic SSI, a smaller square spot of radius 12 was used. All SSIs were constructed and visualized in Python using Matplotlib.

### Section matching

SPOmiAlign is designed around a correspondence-centric alignment paradigm, in which robust dense correspondences form the basis for all subsequent geometric estimation and integration steps.

**Robust dense feature matching.** SPOmiAlign employs RoMa [27] to compute dense pixel-level correspondences between two input sections, supporting both histological images and SSIs. RoMa uses a frozen DINOv2 [29] backbone to extract robust visual features and infers dense, uncertainty-aware correspondences across entire tissue sections via a coarse-to-fine matching strategy. The resulting dense deformation field and per-pixel matchability scores are used for downstream geometric transformation estimation and spatial alignment.

**Edge enhancement.** To improve correspondence selection near tissue boundaries, we introduce an edge enhancement matching strategy that augments RoMa-based correspondence confidence with edge-aware reweighting and spatial uniformization. For an input image with resolution $1152 \times 864$, RoMa generates dense correspondences for all $995{\,}328$ pixels. The original RoMa-based alignment strategy estimates the transformation using only the top $K$ correspondences ranked by confidence (typically $K = 5000$). However, this strategy biases the selected correspondences toward highly textured interior regions, while tissue boundaries, often characterized by weaker visual features, are underrepresented, resulting in suboptimal alignment near tissue edges. To enhance boundary alignment while preserving global alignment performance, we propose an edge enhancement matching strategy that combines edge-aware confidence reweighting with spatial uniformization. To reduce the dominance of strong local textures and emphasize global structural features, we first apply Gaussian smoothing to the grayscale image:


(1) \begin{eqnarray*}& I_{G}(x,y) = (I * G_\sigma)(x,y),\end{eqnarray*}

where $I(x,y)$ denotes the original image and the Gaussian kernel $G_\sigma$ is defined as


(2) \begin{eqnarray*}& G_\sigma(x,y) = \frac{1}{2\pi\sigma^{2}} \exp\left(-\frac{x^{2} + y^{2}}{2\sigma^{2}}\right).\end{eqnarray*}

Following smoothing, a frequency-domain edge weight map is constructed to enhance prominent tissue boundaries while suppressing low-frequency background variations and unstable high-frequency noise. This design emphasizes mid-frequency structural components corresponding to tissue boundaries while suppressing low-frequency background variation and unstable high-frequency noise.

Specifically, a 2D Fourier transform is applied to the smoothed image,


(3) \begin{eqnarray*}& \mathscr{F}(u,v) = \mathrm{FFT2}\big(I_{G}(x,y)\big),\end{eqnarray*}

followed by spectrum centering such that the zero-frequency component is located at the frequency-domain origin. Let $(u,v)$ denote frequency coordinates with respect to the spectrum center, and define the radial frequency distance


(4) \begin{eqnarray*}& D(u,v) = \sqrt{u^{2} + v^{2}}.\end{eqnarray*}

To emphasize boundary-related structural components, a Gaussian high-pass weighting function is applied,


(5) \begin{eqnarray*}& W_{g}(u,v) = 1 - \exp\!\left(-\frac{D^{2}(u,v)}{2\sigma_{\mathrm{val}}^{2}}\right),\end{eqnarray*}

where $\sigma _{\mathrm{val}}$ denotes the frequency-domain cutoff scale (in pixel-frequency units) and is obtained by mapping the dimensionless hyperparameter $\sigma$ to the image size as


(6) \begin{eqnarray*}& \sigma_{\mathrm{val}} = \sigma \cdot \frac{\min(M,N)}{100},\end{eqnarray*}

with $\sigma = 2.0$ fixed in all experiments, yielding $\sigma _{\mathrm{val}} = 0.02 \times \min (M,N)$. Here, $M$ and $N$ denote the image width and height (in pixels), respectively. To suppress unstable responses from extreme high frequencies, a radial exponential decay term is further introduced,


(7) \begin{eqnarray*}& W_{d}(u,v) = \exp\!\left(-\frac{D(u,v)}{\alpha}\right),\end{eqnarray*}

with the decay parameter fixed to $\alpha = 5.0\ \times \min (M,N)$ (i.e. $decay = 5.0$). The final frequency-domain weighting function is obtained by combining both terms multiplicatively,


(8) \begin{eqnarray*}& W(u,v) = W_{g}(u,v)\,W_{d}(u,v).\end{eqnarray*}

The weighted spectrum is then given by


(9) \begin{eqnarray*}& \mathscr{F}^{\prime}(u,v) = \mathscr{F}(u,v)\cdot W(u,v),\end{eqnarray*}

and transformed back to the spatial domain via an inverse Fourier transform,


(10) \begin{eqnarray*}& I_{E}(x,y) = \Re\!\left(\mathrm{IFFT2}\big(\mathscr{F}^{\prime}(u,v)\big)\right),\end{eqnarray*}

where $\Re (\cdot )$ denotes the real component. The magnitude of the reconstructed signal is taken as a continuous edge response map and normalized to the range $[0,1]$. The normalized edge response is first enhanced using a power-law transformation,


(11) \begin{eqnarray*}& E_\gamma(x,y) = E_{\mathrm{norm}}(x,y)^{\gamma},\end{eqnarray*}

with $\gamma = 1.2$. An adaptive global threshold $\tau$ is then determined using Otsu’s method, yielding a binary edge mask


(12) \begin{eqnarray*}& E(x,y) = \begin{cases} 1, & E_\gamma(x,y)> \tau, \\ 0.1, & \mathrm{otherwise}, \end{cases}\end{eqnarray*}

where pixels with $E(x,y)=1$ correspond to prominent tissue boundaries.

Although edge-aware reweighting improves boundary alignment, it can lead to excessive point density along tissue edges. To ensure a globally uniform correspondence distribution, we introduce a spatial uniformization module. This step prevents over-concentration of correspondences along high-contrast boundaries, which could otherwise bias geometric estimation and degrade global alignment stability. The image domain is partitioned into sliding $3 \times 3$ pixel neighborhoods. Within each neighborhood $\Omega _{j}$, only the correspondence with the highest reweighted confidence is retained:


(13) \begin{eqnarray*}& i^{*} = \arg\max_{i \in \Omega_{j}} \tilde{p}_{i}.\end{eqnarray*}

All correspondences remaining within the same neighborhood are discarded. The resulting correspondence subset (top-$K$ correspondences, with $K = 5000$) is then used to estimate the transformation from image $A$ to image $B$.

### Section warping

Depending on the alignment scenario, SPOmiAlign supports rigid, affine, and nonrigid transformations. In practice, a two-stage strategy is employed. An affine transformation is first estimated from the optimized correspondence set to achieve coarse global alignment, followed by nonrigid refinement using a B-spline deformation model, chosen for its flexibility and numerical stability. The estimated transformation is applied to the spatial coordinates of all spots in the source section, yielding warped coordinates that serve as the final aligned spatial representation for downstream analysis.

### Reassignment

Differences in sequencing technologies lead to mismatched spatial resolutions and spot densities across spatial omics datasets, precluding direct spot-wise correspondence after alignment. To enable downstream multiomic integration, we apply a reassignment step to establish one-to-one correspondence between spots. After warping, the section with the higher spatial resolution is designated as the reference. For each reference spot, the molecular profile of its nearest neighboring spot in the source section is assigned based on the Euclidean distance. To correct for resolution-induced bias, where a single source spot may be reassigned to multiple reference spots, the reassigned profiles are normalized by the number of assignments per source spot. To avoid erroneous reassignment in nonoverlapping regions, a distance-based filtering criterion is applied. Specifically, reassignment is performed only when the nearest-neighbor distance is below a global threshold derived from the average inter-spot distances of the two sections. Reference spots without a valid neighboring source spot are excluded from subsequent integrative analyses.

### Vertical multiomic integration

After section matching and reassignment, multiomic sections were brought into one-to-one spot correspondence, yielding a unified set of spatial locations shared across modalities. Vertical multiomic integration was performed using SpatialGLUE [30] to jointly embed multiomic molecular profiles in a shared latent space. Subsequently, Leiden clustering was applied to the integrated embeddings to identify spatial domains. To determine the optimal clustering resolution, we evaluated clustering hierarchies using Clustree [31], which enables visualization of cluster stability across resolutions. Based on Clustree analysis, the optimal number of clusters was selected for spatial domain identification and downstream refinement.

### Differential analysis of multiomic data

Differential analyses of ST, SP, and SM were performed between the IML and OML subregions identified within five groups. For ST and SP, normalized gene expression and protein abundance matrices were extracted from the integrated AnnData object. Differential tests were conducted using the Wilcoxon rank-sum test (implemented in Scanpy) to ensure methodological consistency [32]. Log-fold changes (logFC) were calculated based on the difference in mean expression or abundance between subregions. For SM, normalized metabolite intensity profiles were compared using the Wilcoxon rank-sum test to account for non-Gaussian signal distributions. For all modalities, the $P$-values were adjusted for multiple tests using the Benjamini–Hochberg (BH) procedure. Features with an adjusted $P$-value <0.1 and logFC >0.25 were considered statistically significant. Differentially upregulated and downregulated features were extracted for downstream integration.

### Metabolite annotation

Differentially abundant spatial metabolomic features were annotated by matching observed m/z values to the Human Metabolome Database (HMDB) [33, 34]. The matching was performed within a mass tolerance window of 3 ppm. Putative identities were assigned based on molecular formula and monoisotopic mass. To improve coverage, multiple adduct forms in negative ionization mode were considered, including [M-H]-, [M+Cl]-, [M+FA-H]-, and [M+Ac-H]-. Annotated metabolites were classified into superclass, class, and subclass categories according to the HMDB taxonomy and grouped into lipid and non-lipid categories for interpretation of the pathway.

### Integrated analysis of ST, SP, and SM

**Integration of ST-SP.** We adopted a stepwise integration strategy centered on transcription–protein concordance to identify molecular programs supported across modalities. To identify robust molecular programs supported across the transcriptional, proteomic, and metabolic layers, we adopted a stepwise integration strategy centered on the concordance between spatially variable genes (SVGs) and spatially variable proteins (SVPs). The ST and SP datasets were first aligned based on shared gene symbols and independent differential analyses were performed to identify the features regulated between the inner (IML) and outer (OML) molecular layers. We initially prioritized gene–protein pairs that show significant positive associations using Spearman’s rank correlation analysis (Spearman’s $\rho>0.1$, FDR $<0.05$). To further ensure robust regulatory consistency, a coherence filtering threshold of 0.45 was applied to retain features that exhibit strong directional agreement, thus defining a high-confidence ”ST–SP concordant signature.” These features were ranked according to their combined differential signals and visualized using heat maps.

**ST–SP–SM integration.** Building upon this validated transcriptomic–proteomic axis, SM data were subsequently integrated to characterize the specific metabolic states and lipid microenvironments associated with these coordinated molecular programs. Spatial spots were ordered based on the ranked expression scores of the ST–SP concordant signature. Normalized profiles for genes, proteins, and metabolites were extracted from spatially aligned coordinates. Spearman’s rank correlation analysis was performed to identify metabolite–gene and metabolite–protein pairs that show a concordant spatial variation (Spearman’s $\rho>0.2$, FDR $<0.05$). Significant associations were summarized to define preferentially enriched metabolite classes in transcriptionally and proteomically defined spatial domains (IML versus OML).

### Functional enrichment analysis

Functional enrichment was performed to interpret biological programs that drive sublayer stratification. Differentially expressed genes (ST) and proteins (SP) were independently analyzed using Enrichr [35]. The queries included the Gene Ontology (GO) Biological Process (2025), Reactome Pathways (2024), SynGO (2024), and the MGI Mammalian Phenotype (Level 4, 2024), together with reference datasets of brain cells including the Allen Brain Atlas (10x scRNA 2021) and Azimuth (2023). Enrichment analysis was further applied to the ST–SP concordant gene set to identify pathways robustly supported by both the transcriptional and proteomic layers. Statistical significance was assessed using Fisher’s exact test with BH correction. Terms with an adjusted $P$-value ¡.05 were considered significant. The results were visualized using ranked bar plots to highlight consistent biological processes.

### Metrics for evaluating registration to the common coordinate framework

**Full width at half maximum.** To assess spatial registration precision at the molecular level, we quantified the FWHM of marker-specific spatial distributions after alignment to the Allen CCF. For a given molecular marker, we first estimate its spatial density across all spots in the aligned section. For each spot, we then computed the shortest Euclidean distance to the boundary of the corresponding CCF anatomical region obtained after annotation transfer. The distribution of these distances was summarized as a 1D density function. Let $d$ denote the distance to the boundary of the corresponding CCF region, and let $f(d)$ denote the estimated density function of $d$. The FWHM was defined as the width of the distance distribution at half of its maximum density value, i.e.


(14) \begin{eqnarray*}& \mathrm{FWHM} = d_{2} - d_{1},\end{eqnarray*}

where $d_{1}$ and $d_{2}$ satisfy


(15) \begin{eqnarray*}& f(d_{1}) = f(d_{2}) = \tfrac{1}{2}\max_{d} f(d),\end{eqnarray*}

with $d_{1} < d_{2}$. A smaller FWHM indicates that marker-positive spots are more closely localized around the corresponding anatomical boundaries of the CCF, reflecting the higher spatial registration accuracy between the spatial omics data and the CCF.

**Dice coefficient.** To evaluate region-level alignment accuracy, we computed the Dice coefficient between manually annotated brain regions in the spatial omics data and the corresponding brain regions assigned by SPOmiAlign after annotation retrieval. For a given brain region, let $A$ denote the set of spots labeled by manual annotation and $B$ denote the set of spots assigned to the corresponding CCF region by SPOmiAlign. The Dice coefficient is defined as


(16) \begin{eqnarray*}& \mathrm{Dice}(A, B) = \frac{2\,|A \cap B|}{|A| + |B|}.\end{eqnarray*}

A larger Dice coefficient indicates greater overlap between ground-truth annotations and CCF-retrieved annotations, corresponding to higher registration accuracy.

### Metrics for evaluating spatial multiomic alignment

**Cell type-level PCC and cosine similarity.** In the evaluated datasets, cell type annotations were available for section S1 but absent for section S2. To enable a cell type–based alignment assessment, section S2 was manually annotated. To ensure consistency of cell-type definitions across sections, annotations in S1 were further curated by merging fine-grained subclasses into broader categories shared between S1 and S2. For each matched cell type, we assessed the spatial compositional correspondence between modalities after alignment. Specifically, the PCC and cosine similarity were calculated between the representations of the cell-type in the matched spots in S1 and the aligned spots in S2. Higher PCC and cosine similarity values indicate stronger agreement in the spatial distributions of the cell-type between modalities, reflecting improved multiomic alignment.

**Molecular-level Bivariate Moran’s *I* for spatial co-localization.** In spatial multiomic data, different modalities are characterized by distinct molecular measurements, such as gene expression levels in ST and metabolite intensities in SM. To evaluate multiomic alignment, it is therefore essential to quantify the spatial concordance of co-localized biomarkers across omics. Previous single-modality studies commonly employ univariate Moran’s *I* to measure spatial autocorrelation of molecular features. In the multimodal setting, we extend this concept by computing bivariate Moran’s *I*, which captures the spatial association between biomarkers from two different modalities. Formally, let $x_{i}$ and $y_{j}$ denote the values of two biomarkers from different modalities in spatial locations $i$ and $j$, respectively, and let $w_{ij}$ denote the spatial weight matrix encoding neighborhood relationships. We constructed the spatial weight matrix based on the spatial coordinates of the SM section using a $K$-nearest neighbors (KNN) graph. We set the number of neighbors to $k = 6$, and applied row-standardization to the weight matrix before computing the statistic. We then calculated bivariate Moran’s $I$ using this KNN-based spatial weight matrix. The bivariate Moran’s *I* is defined as


(17) \begin{eqnarray*}& I_{xy} = \frac{n}{S_{0}} \frac{\sum_{i}\sum_{j} w_{ij}(x_{i} - \bar{x})(y_{j} - \bar{y})} {\sqrt{\sum_{i}(x_{i} - \bar{x})^{2} \sum_{j}(y_{j} - \bar{y})^{2}}},\end{eqnarray*}

where $n$ is the total number of spatial locations, $\bar{x}$ and $\bar{y}$ denote the mean values of the two biomarkers, and


(18) \begin{eqnarray*}& S_{0} = \sum_{i}\sum_{j} w_{ij}.\end{eqnarray*}

A higher value of the bivariate Moran’s *I* indicates stronger spatial co-localization between biomarkers across omics, corresponding to a more accurate multiomic alignment.

### Metrics for evaluating vertical multiomic integration and spatial domain clustering

In addition, clustering performance was evaluated using standard clustering metrics, including ARI, NMI, silhouette score, Calinski–Harabasz index, homogeneity, completeness, and V-measure. These metrics were calculated based on spot-matched labels after alignment and reassignment.

## Results

### Overview of SPOmiAlign

**Figure 1:**
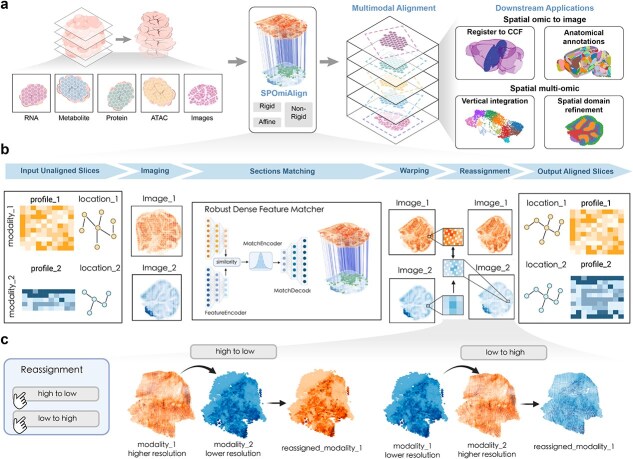
Overview of SPOmiAlign for modality-agnostic spatial multimodal alignment. a End-to-end application workflow. b Algorithmic pipeline of SPOmiAlign. c Schematic illustration of the reassignment module.

SPOmiAlign is a new modality-agnostic spatial omics alignment framework. Given sections from arbitrary spatial modalities, SPOmiAlign performs cross-section alignment and supports downstream spatial multimodal analysis in two major settings: spatial omic-to-image and spatial multi-omic integration (Fig. 1a). In the spatial omic-to-image setting, SPOmiAlign enables the registration of spatial omics sections into a CCF and supports automated transfer of anatomical region annotations. In the spatial multi-omic setting, it facilitates vertical integration and spatial domain refinement by establishing spatial correspondence across modalities.

The SPOmiAlign framework comprises four major components: imaging, section matching, warping, and reassignment (Fig. 1b). In the imaging module, spatial omics profiles are converted into image-like representations to enable cross-modality comparison. For spatial omics sections with paired imaging data, including histological or fluorescence images, the paired images are directly used for downstream alignment. For sections lacking paired images, molecular profiles are transformed into SSIs, which preserve spatial organization and enable subsequent image-based registration.

To align two sections, SPOmiAlign leverages RoMa [27], which builds on DINOv2-based [29] visual embeddings and integrates a Gaussian process encoder with a transformer-based decoder to identify dense correspondences across modalities. Based on these matched correspondences, geometric transformations are estimated and applied through a dedicated warping module, supporting rigid, affine, and nonrigid transformations to correct both global orientation differences and local geometric distortions.

Because differences in spatial resolution and sampling density across modalities often prevent one-to-one correspondence between spots even after geometric alignment, SPOmiAlign further incorporates a reassignment module to reconcile these discrepancies. This module enables one-to-one spot correspondence across sections and is particularly important for vertical downstream integration tasks that require spatially matched observations across datasets. Two reassignment directions are supported: high-resolution to low-resolution and low-resolution to high-resolution. This flexibility allows users to choose the reassignment direction according to the relative resolutions of the modalities and their specific analysis goals (Fig. 1c).

**Figure 2:**
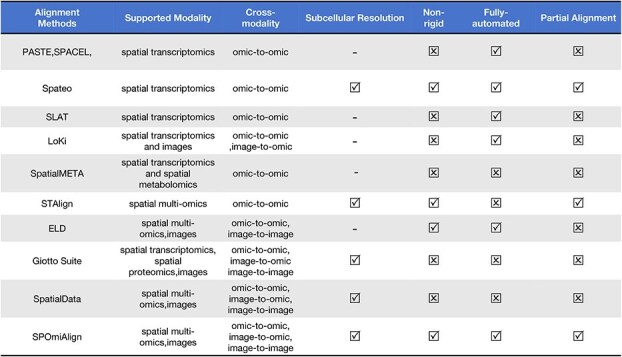
Summary table comparing SPOmiAlign with 10 representative spatial omics slice alignment methods across multiple aspects, including supported modality, cross-modality type, support for subcellular-resolution sections, support for nonrigid transformation, degree of automation, and support for partial-overlap alignment.

Furthermore, to clearly position SPOmiAlign relative to existing general-purpose spatial toolkits and spatial alignment methods, Fig. 2 provides a summary comparison. This summary table compares SPOmiAlign with 10 existing spatial omics slice alignment methods. The comparison includes multiple aspects, including supported modality, cross-modality type, whether the method supports subcellular-resolution sections, whether it supports nonrigid transformation, whether it is fully automated, and whether it supports partial-overlap alignment. This summary also clarifies the types of data to which SPOmiAlign can be applied.

### SPOmiAlign enables registration of spatial omic sections to the common coordinate framework and anatomical annotation retrieval

Accurate registration of spatial omics data in CCF is critical for interpreting molecular patterns within their anatomical context. However, due to the challenges posed by cross-modal heterogeneity (spatial omics to image-based CCF), partial spatial overlap, and nonrigid tissue deformations, most existing approaches still rely heavily on manual landmark annotation [36, 37]. SPOmiAlign enables fully automated registration of experimental sections in an anatomical atlas, such as the Allen Brain Atlas [38], thus supporting standardized anatomical annotation at single-spot resolution.

**Figure 3:**
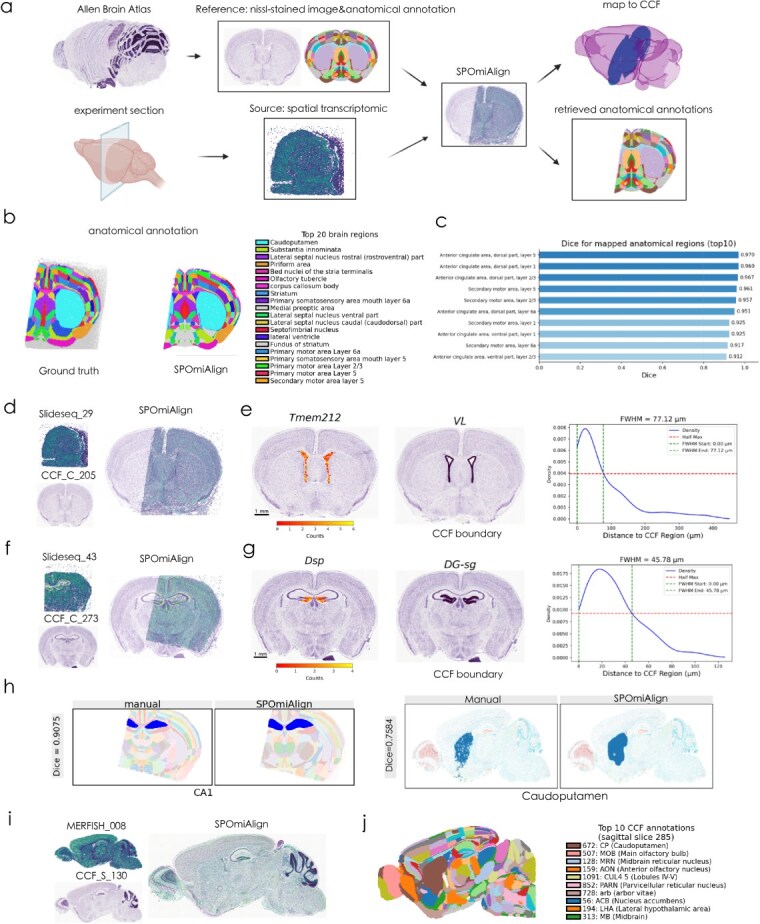
SPOmiAlign enables registration of spatial omics to image-based CCF and anatomical annotation retrieval. a Schematic overview of automated spatial-omics registration to the CCF using SPOmiAlign. b Comparison of anatomical annotations obtained by SPOmiAlign and ground truth for a representative section, with the top 10 brain regions ranked by area shown in the legend. c Dice similarity coefficients for the top 10 brain regions with the highest annotation agreement between SPOmiAlign and ground truth. d,f Overlay of Slide-seq mouse brain coronal sections 29 (d) and 43 (f) with their matched CCF Nissl-stained reference sections after alignment by SPOmiAlign. e,g Distance density distributions between enriched marker-gene-expression spots and the corresponding CCF region boundaries for Slide-seq sections 29 (e) and 43 (g). h Dice coefficient comparison between SPOmiAlign-derived and ground-truth annotations for two representative regions: CA1 in Slide-seq section 43 (left) and Caudoputamen in MERFISH sagittal section 008 (right). i Overlay of MERFISH mouse brain sagittal section 008 with the matched CCF reference section after alignment by SPOmiAlign. j Anatomical annotation map assigned to MERFISH section 008 by SPOmiAlign, with the top 10 annotated regions by area shown in the legend (CCF annotation section at $z=285$).

To evaluate the registration performance of SPOmiAlign, we analyzed two 3D mouse brain spatial transcriptomic datasets generated using distinct platforms: a sequencing-based Slide-seq coronal dataset and an imaging-based MERFISH sagittal dataset [36, 37]. Specifically, SPOmiAlign maps experimental ST sections to the Allen Brain Atlas by first identifying the Nissl-stained image with the anatomically corresponding section of the atlas and aligning the experimental section with this reference (Fig. 3a). Following registration, each spatial spot is assigned to a CCF coordinate, from which standardized anatomical annotations are retrieved.

For the Slide-seq dataset, two sections exhibiting clear anatomical structures were selected for evaluation. For ID 29, visual inspection confirmed strong spatial concordance between the corresponding Nissl-stained images and the aligned ST sections (Fig. 3d, f). Manual anatomical annotations from the Allen Brain Atlas (Method) were used as ground truth to benchmark the anatomical labels generated by SPOmiAlign. Since existing alignment methods often struggle to simultaneously support spatial omics–to–image alignment, handle partial spatial overlap, and operate in a fully automated manner, manually curated annotations provide a reliable reference standard for objective comparison. At a qualitative level, the automatically assigned region annotations closely recapitulated the spatial organization of the manually curated labels across ID 29 coronal sections (Fig. 3b, ID 43 in Supplementary Fig. 1b). The registration accuracy was quantified using the Dice coefficient, which measures the spatial overlap between the corresponding anatomical region masks. Across the top 10 regions with the highest alignment accuracy, the Dice coefficients exceeded 0.91 for Slide-seq section ID 29 (Fig. 3c, more regions in Supplementary Fig. 1c), with particularly strong concordance observed in fine-grained cortical regions such as the anterior cingulate area and the secondary motor area, highlighting the precision of SPOmiAlign in aligning anatomically detailed structures. Representative regions, including the cornu ammonis area 1 (CA1; Slide-seq ID 43, Fig. 3h), the caudoputamen, and the piriform cortex (Slide-seq ID 29, Supplementary Fig. 1a), each achieved Dice coefficients greater than 0.72.

To assess biological concordance beyond geometric overlap, we examined genes with well-established region-specific expression patterns. Consistent with manual annotations, *Dsp* was enriched in the dentate gyrus subgranular zone (DG-sg), while *Tmem212* showed preferential expression in the ventral lateral thalamic region. The full expression variation across the tissue is shown in (Supplementary Fig. 1e) showing that these two genes are specifically enriched at the CCF boundary, rather than broadly expressed in nearby surrounding regions. For each gene, we quantified the spatial deviation between high-expression spots and atlas-defined region boundaries by measuring the full width at half maximum (FWHM) of the distance distribution [36]. Across enriched genes, the FWHM values ranged from 40 to 70 $\mu$m, comparable with the precision typically achieved by expert manual alignment (Fig. 3e, g, Supplementary Fig. 1d), indicating that region-specific molecular signals remain spatially coherent after SPOmiAlign mapping.

In addition, we evaluated SPOmiAlign on the MERFISH sagittal dataset. Despite substantial differences in measurement modality, imaging resolution, and spatial organization, SPOmiAlign produced coherent anatomical mappings (Fig. 3i, j). The Dice scores for representative regions (Fig. 3h) demonstrated a high agreement with the atlas annotations, underscoring the robustness of SPOmiAlign across different technologies.

Finally, we evaluated the cross-technology alignment performance in ST by aligning a MERFISH slice (092) to a Slide-seq slice (57). The results showed that SPOmiAlign is capable of cross-technology alignment of brain sections (Supplementary Fig. 2a), with improved spatial correspondence for the marker genes *Fibcd1* and *Chrm3* in the CA1 and CA3 regions (Supplementary Fig. 2b). Furthermore, we tested the robustness of SPOmiAlign on a more challenging task. When aligning two cross-modality sections with only a small overlapping region, we found that the correspondences identified by SPOmiAlign were much less accurate (Supplementary Fig. 2c). These results indicate a limitation of SPOmiAlign, namely that its performance degrades in cross-modality partial-overlap alignment scenarios with very limited shared tissue area.

Together, these results demonstrate that SPOmiAlign provides robust and fully automated alignment for challenging spatial omic-to-image registration tasks, simultaneously handling cross-modal discrepancies, partial spatial overlap, and complex nonrigid tissue deformations. This robustness allows SPOmiAlign to generalize across spatial omics platforms and imaging modalities without modality-specific tuning or manual intervention.

### SPOmiAlign enables image-to-image registration

**Figure 4:**
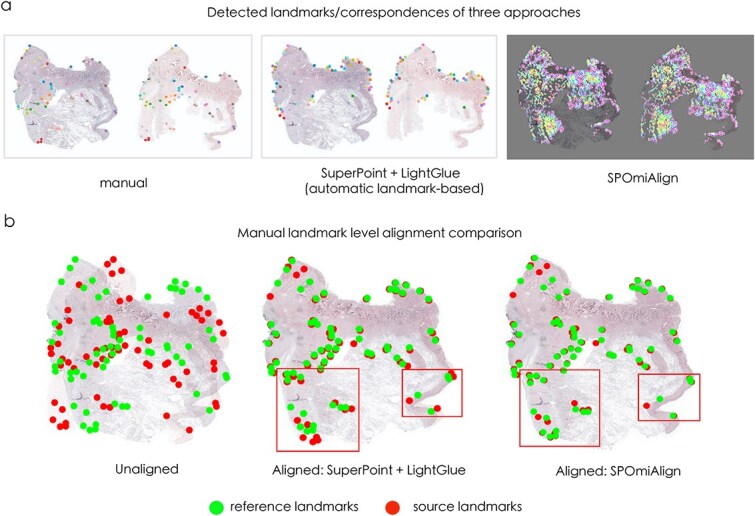
SPOmiAlign enables robust image-to-image registration on the ANHIR benchmark. a Comparison of correspondences identified by manual landmarks, the automatic landmark detection method SuperPoint, and SPOmiAlign on representative ANHIR image pairs. b Quantitative evaluation of alignment performance using manually annotated landmarks as an independent reference.

To further validate the image-to-image registration component underlying SPOmiAlign, we evaluated its performance on the ANHIR benchmark dataset, a public reference standard for nonrigid histological image registration [39]. ANHIR comprises serial or adjacent histological sections with substantial nonrigid deformations and staining variability, and each image pair is annotated with manually defined landmarks. We therefore first compared three types of detected correspondences: manual landmarks, the automatic landmark detection method SuperPoint [24], and the correspondences identified by SPOmiAlign. The results showed that landmarks detected by the automatic landmark-based method were less globally distributed and tended to miss correspondences in some regions (Fig. 4a). In contrast, the correspondences identified by SPOmiAlign were more globally distributed across the tissue, which is more beneficial for subsequent alignment.

We next assessed the alignment performance of SPOmiAlign using the manual landmarks as an independent reference. Before alignment, corresponding manual landmarks were often spatially far apart between the two sections, whereas after alignment their distances were substantially reduced. Smaller post-alignment distances indicate better alignment quality. Compared with SuperPoint, SPOmiAlign achieved better performance with respect to the spatial consistency of manual landmarks, supporting that its globally distributed correspondences provide an advantage over conventional automatic landmark-based methods (Fig. 4b).

Moreover, across diverse tissue types, disease conditions, and staining protocols, SPOmiAlign consistently achieved accurate and efficient alignment, demonstrating robust performance under challenging nonrigid distortions (Supplementary Fig. 4). Together, these results show that SPOmiAlign achieves competitive performance for nonrigid image-to-image registration, further complementing its effectiveness in histology-guided spatial multiomic alignment.

### SPOmiAlign enables histology image-guided spatial multiomic alignment

**Figure 5:**
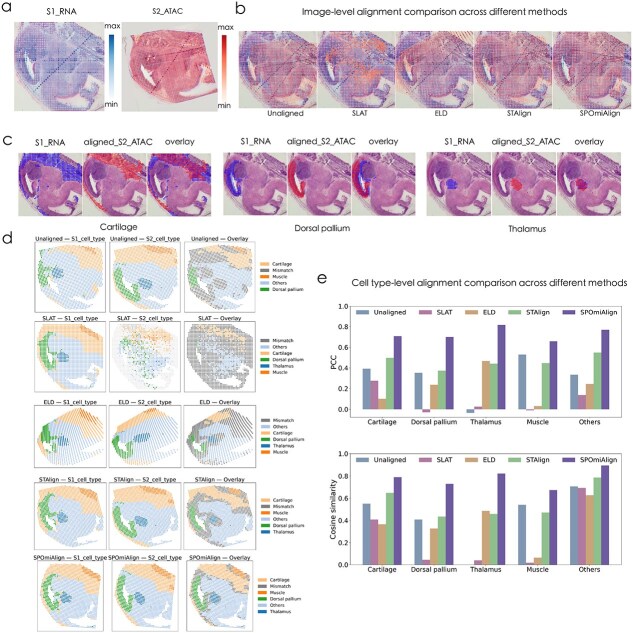
SPOmiAlign enables robust histology image-guided spatial multiomic alignment. a MISAR-seq mouse brain spatial multiomic dataset, where Sample 1 (S1) and Sample 2 (S2) were profiled by ST and spatial ATAC-seq, respectively. b Overlay of the S1 RNA-derived SSI and the aligned S2 ATAC-derived SSI on the histology background, shown for the unaligned data and after alignment with SLAT, ELD, STAlign, and SPOmiAlign. c Representative examples of cell-type overlap between S1 and aligned S2 following SPOmiAlign for cartilage, dorsal pallium, and thalamus. d Visualization of cell-type annotations for S1 (left column), aligned S2 (middle column), and their overlay (right column) under different alignment methods; the black boxes indicate local regions with mismatched spots between corresponding cell types. e Quantitative evaluation of spatial concordance between S1 and S2 across cell types using PCC (top) and cosine similarity (bottom) for different alignment methods.

To evaluate the performance of SPOmiAlign for spatial multiomic alignment, we analyzed a multiomic mouse brain dataset consisting of ST (Sample 1, S1) and spatial ATAC-seq (Sample 2, S2) generated from two independent mice [4]. As each section was associated with a paired H&E histology image, these images were used as intermediate references to allow image-guided alignment between the two spatial omics sections. To systematically evaluate alignment robustness under increasing deformation, we applied synthetic geometric perturbations—including rotation, translation, and scaling—to the spatial coordinates of S2, creating controlled inter-section discrepancies for benchmarking under increasingly challenging alignment conditions (Fig. 5a).

To compare the alignment performance of SPOmiAlign against representative methods capable of aligning spatial multimodal tissue sections, SPOmiAlign was compared with SLAT [11], an automated multimodal spatial omics matching method, ELD [20], an automated multimodal image registration method, and STAlign [15], a ST alignment approach, as well as unaligned sections. The alignment quality was visualized using overlap of aligned SSIs, rendered on the H&E-stained image of section S1 as background, demonstrating that SPOmiAlign achieved qualitatively accurate alignment, particularly in tissue boundaries (Fig. 5b). All methods were evaluated in identical preprocessing and perturbation settings.

Here, to evaluate the performance of spatial multiomic alignment, we adopted spatial correlation metrics at the cell-type level, including the Pearson correlation coefficient (PCC) and cosine similarity. Manual cell-type annotations from the ST section (S1) and clustering-based annotations from the ATAC-seq section (S2) were harmonized to ensure correspondence across datasets, with unmatched labels grouped into a category “other.” Visual inspection of representative cell types revealed qualitatively accurate overlap after alignment with SPOmiAlign (Fig. 5c). Consistently, the number of mismatch spots, defined as spatially inconsistent cell-type assignments, was markedly reduced compared with SLAT, ELD, and STAlign (Fig. 5d). Across all corresponding cell types, SPOmiAlign consistently outperformed SLAT, ELD, and STAlign, exhibiting markedly higher PCC and cosine similarity values, indicative of superior spatial concordance (Fig. 5e).

To assess runtime for different alignment methods, we evaluated computational efficiency by repeating the alignment procedure three times on a high-resolution alignment dataset, MERFISH to Slide-seq. In contrast to ELD, SLAT, and STAlign, which typically require minutes to hours for alignment, SPOmiAlign reduces computational time by two to three orders of magnitude (Supplementary Fig. 2d). To assess scalability for high-resolution spatial datasets, we further evaluated computational efficiency by repeating the alignment procedure five times using SPOmiAlign. Runtime analysis demonstrated that SPOmiAlign consistently achieves alignment in seconds ($\sim$10 s per run, Supplementary Fig. 3). This substantial speedup highlights the scalability of SPOmiAlign and underscores its suitability for future spatial omics datasets with rapidly increasing spatial resolution.

### SPOmiAlign enables kidney cancer spatial omic-to-omic alignment

To evaluate the performance of SPOmiAlign for spatial multiomic alignment beyond brain tissue, we analyzed a kidney cancer multiomic dataset consisting of paired ST and SM sections [21]. As only one section was associated with a paired H&E histology image, we first generated SSIs for each omics modality and then performed alignment through the SSI representations.

**Figure 6:**
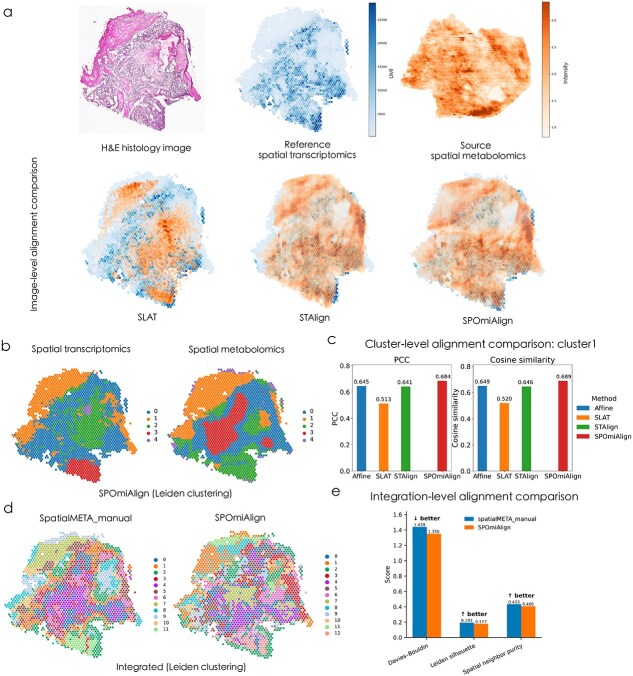
SPOmiAlign enables kidney cancer spatial omic-to-omic alignment. a Overlay of aligned SSIs from paired ST and SM sections, comparing SPOmiAlign, SLAT, STAlign, and manual affine transformation. b Leiden clustering results for the two modalities before integration, with five clusters identified in each modality. c Quantitative evaluation of alignment performance using PCC and cosine similarity within cluster 1, showing the best performance for SPOmiAlign. d Leiden clustering of the integrated multiomic data generated using the same SpatialMETA-based integration workflow for both SPOmiAlign-aligned and SpatialMETA-aligned sections, with clustering resolution in 0.8. e Quantitative comparison of integration performance between SPOmiAlign and SpatialMETA after downstream integration.

To compare the alignment performance of SPOmiAlign against representative methods capable of aligning spatial multimodal tissue sections, we benchmarked SPOmiAlign against SLAT [11], STAlign [15], and manual affine transformation. Image-level alignment quality was visualized by overlaying the aligned SSIs, showing that SPOmiAlign achieved qualitatively accurate alignment, particularly at tissue boundaries (Fig. 6a).

To further evaluate spatial multiomic alignment performance, we adopted cluster-level spatial correlation metrics, including the PCC and cosine similarity. Because this dataset did not contain manual cell-type annotations, we performed Leiden clustering separately for the two omics modalities and identified five clusters in each omic (Fig. 6b). We observed that cluster 1 represented a potentially corresponding cluster shared between the two modalities. We therefore calculated PCC and cosine similarity within cluster 1. Among the four compared methods, SPOmiAlign achieved the highest PCC and cosine similarity, indicating better alignment performance (Fig. 6c).

In addition, because SpatialMETA [21] supports alignment between SM and ST sections, we further included SpatialMETA in the benchmark. We note that SpatialMETA requires a manual affine transformation step as part of its alignment pipeline. Because SpatialMETA is also an integration algorithm, for this comparison, we evaluated performance at the integration level. To ensure fairness, we first performed downstream multiomic integration for both SPOmiAlign-aligned and SpatialMETA-aligned sections using the same SpatialMETA-based integration workflow. Leiden clustering of the integrated data identified multiple clusters at a clustering resolution of 0.8 (Fig. 6d), after which we calculated metrics to assess clustering performance after integration. The results showed that SPOmiAlign achieved performance comparable with SpatialMETA, despite SpatialMETA requiring manual intervention during alignment. These findings further demonstrate the ability of SPOmiAlign to align multiomic tissue sections beyond brain tissue, including datasets without clear spatial anatomical organization (Fig. 6e).

### SPOmiAlign supports spatial tri-omic alignment and facilitates spatial multiomic integration analysis

Recent advances in spatial multiomics have expanded beyond two omics toward tri-omics, enabling more comprehensive characterization of tissue organization. Accurate alignment of multiomic sections into a shared coordinate system, while preserving one-to-one correspondence between spatial observations, is a prerequisite for meaningful integrative multiomics analysis. However, increasing the complexity of the modality introduces substantially greater challenges for accurate spatial alignment across datasets.

**Figure 7:**
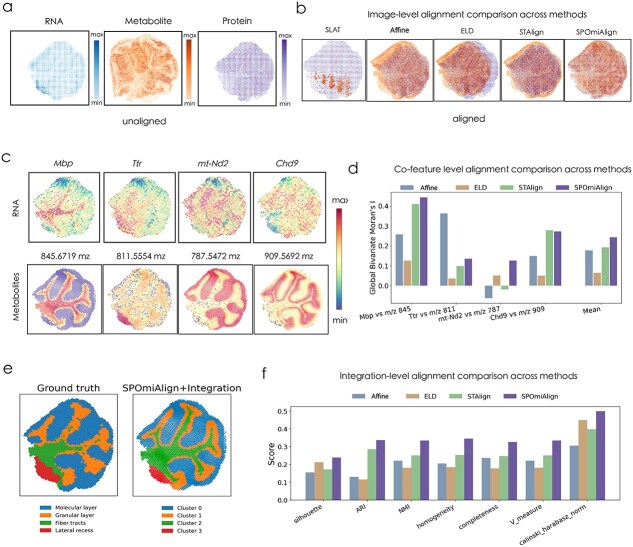
SPOmiAlign supports spatial tri-omic alignment and facilitates spatial multiomic integration. a SSIs of three adjacent mouse brain sections profiled by ST, SM, and SP before alignment, shown separately for each modality. b Overlay of aligned SSIs produced by SLAT, affine transformation, ELD, STAlign, and SPOmiAlign, illustrating spatial correspondence across the three omics layers after alignment. c Spatial distribution maps of four representative cross-modal biomarker pairs showing spatial co-localization between transcriptomic and metabolomic features: *Mbp* with m/z 845.6719, *Ttr* with m/z 811.5554, *mt-Nd2* with m/z 787.5472, and *Chd9* with m/z 909.5692. d Bivariate Moran’s $I$ values for the four biomarker pairs after alignment by affine transformation, ELD, STAlign, and SPOmiAlign, together with the mean Moran’s $I$ for each method. e Comparison between anatomical ground-truth regions (left) and clustering results obtained after SPOmiAlign-based alignment followed by downstream integration (right), showing recovery of major cerebellar spatial domains. f Quantitative evaluation of integration quality using multiple standard metrics for four alignment methods, followed by integration with SpatialGLUE.

The tri-omic alignment performance of SPOmiAlign was evaluated on a mouse brain spatial tri-omic dataset comprising three adjacent tissue sections profiled using different technologies: ST generated by MAGIC-seq, SP measured by PLATO, and SM acquired via MALDI-MSI [3]. The ST section served as the reference, while the SP and metabolomics sections aligned with the transcriptomic coordinate system. In the absence of paired imaging data, all spatial omics sections were converted to SSIs for alignment. Before alignment, pronounced spatial discrepancies were observed between the SSIs of the three modalities, most notably in the SM section, which exhibited substantial positional offsets relative to the other two modalities (Fig. 7a). Consequently, an initial affine transformation (rotation and scaling; referred to as affine) was applied to the metabolomics section prior to alignment, serving as an identical preprocessing step for all of the compared methods.

Benchmarking against SLAT, STAlign, ELD, and the affine transformed baseline demonstrated that SPOmiAlign achieved consistently improved alignment, with enhanced correspondence at tissue boundaries and finer anatomical structures (Fig. 7b), benefiting from the correspondence-driven matching strategy (Methods). Quantitative evaluation was performed using four pairs of spatially co-localized biomarkers previously reported to exhibit consistent spatial distributions across ST and SM modalities (Fig. 7c). The precision of the alignment was quantified using the bilinear Moran’s I statistic, which measures spatial co-localization between two variables. Across all biomarker pairs, SPOmiAlign yielded the highest mean bilinear Moran’s I values (Fig. 7d), indicating superior spatial concordance relative to comparative methods.

To assess whether improved alignment results in improved downstream multiomic integration, integrative analysis was performed on the aligned tri-omic dataset using an integration approach SpatialGLUE [30]. Because ST data included cell-type annotations, integration quality was evaluated by comparing clustering results after using SPOmiAlign with transcriptomic annotations. When clustering into four domains, the integrated data exhibited strong concordance with known cell-type annotations, while additionally revealing fiber tract structures that were not clearly identifiable in unimodal transcriptomics (Fig. 7e). Across multiple standard integration quality metrics, including normalized mutual information (NMI) and adjusted Rand index (ARI), SPOmiAlign-based alignment consistently outperformed STAlign, ELD and affine aligned datasets, demonstrating that accurate spatial alignment is critical for high-quality multiomic integration (Fig. 7f).

### SPOmiAlign enables the discovery of fine-grained molecular stratification within the cerebellar molecular layer

**Figure 8:**
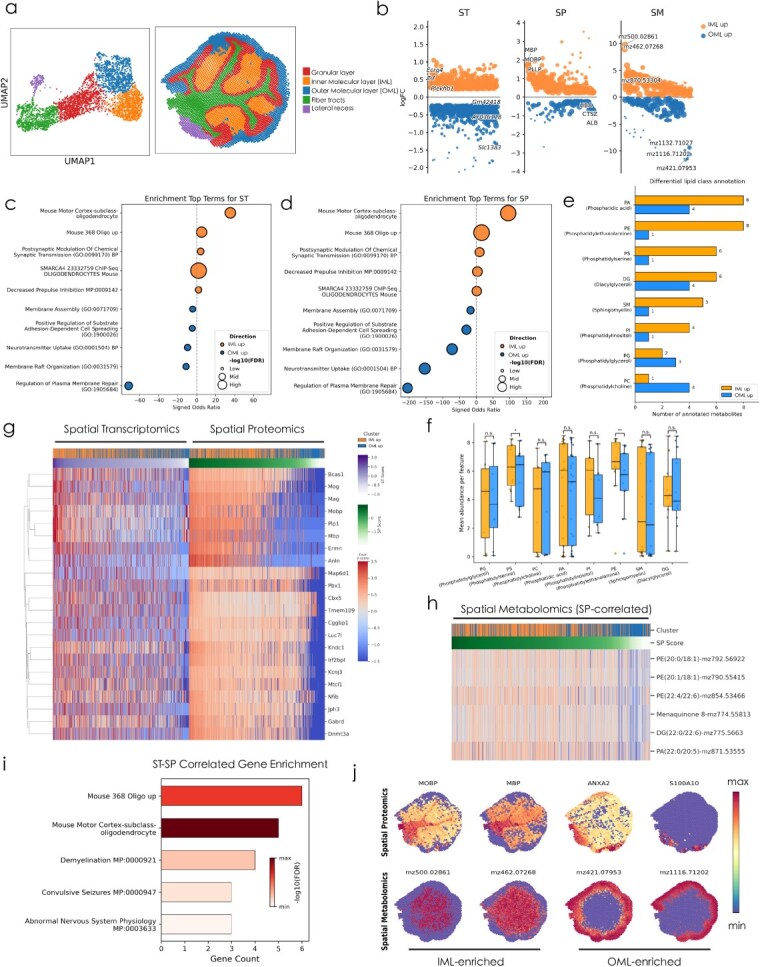
Spatial multiomic dissection of molecular layer substructure in the mouse cerebellum. a UMAP embedding and spatial projection identify stable inner molecular layer (IML) and outer molecular layer (OML) subdivisions. b Differential scatter plots compare ST, SP, and SM features between IML and OML, with representative significant features labeled. c Shared pathway enrichment analysis summarizes biological programs associated with IML-up and OML-up ST signatures. d Shared pathway enrichment analysis summarizes biological programs associated with IML-up and OML-up SP signatures. e Differential lipid class annotation of spatially variable metabolites (SVMs) shows the number of annotated metabolites assigned to each lipid class in the IML-enriched and OML-enriched sets. f Boxplots compare mean intensities of lipid classes between IML and OML using a paired Student’s t-test, with significance codes indicated in the figure. g Heatmap shows jointly regulated SVG-SVP pairs identified from integrated ST-SP analysis, ordered by decreasing mean expression across spatial spots and restricted to positively correlated pairs. h Heatmap shows annotated metabolites correlated with protein signatures from SVG-SVP concordant pairs, with columns representing spatial spots ordered by cluster annotation and rows representing correlated annotated metabolites. i Functional enrichment analysis summarizes biological processes associated with concordant SVG-SVP signatures. j Spatial maps of representative proteins and metabolites identified from integrated multiomic analyses confirm sublayer-specific patterns, with proteomic features shown in the upper row, metabolomic features in the lower row, IML-enriched features in the left columns, and OML-enriched features in the right columns.

Building on the alignment and integration benchmarks above, we next evaluated whether SPOmiAlign could resolve substructures within the cerebellar molecular layer that were not apparent under the original annotation. The partition comprising five groups was supported by consistent cluster connectivity across adjacent resolutions in Clustree and by concordant modality-specific signals after alignment (Fig. 8a, Supplementary Fig. 5a) [31]. At this resolution, the molecular layer was subdivided reproducibly into two spatial sublayers, here termed the inner molecular layer (IML) and the outer molecular layer (OML), while other anatomical regions, including the granular layer, fiber tracts, and lateral recess, remained consistent with the original annotation [3]. This subdivision was robust across adjacent resolutions and supported by concordant patterns across multiple molecular modalities.

Differential analysis of ST and SP, together with enrichment analysis restricted to pathways detected in both modalities, revealed marked molecular divergence between the two sublayers (Fig. 8b–d). The IML showed a strong enrichment of myelin-associated genes and proteins, including *MOBP* and *MBP*, as well as programs associated with oligodendrocytes, consistent with the established roles of mature oligodendrocytes in maintenance and axonal support (Fig. 8j, Supplementary Fig. 6a) [40, 41]. Concurrently, the IML exhibited elevated expression of neurofilament proteins (*NEFH, NEFL, NEFM*; Supplementary Fig. 6b). Given that neurofilaments are ubiquitous structural components of neuronal cytoskeletons, their higher signal in the IML is consistent with an increased local axonal content. Together with oligodendrocyte-enriched transcriptional and proteomic programs, this pattern supports a spatial association between axon-rich compartments and glial programs within the deep molecular layer [42].

In contrast, OML was characterized by the enrichment of pathways related to membrane organization, assembly, and lipid turnover, including annexin-associated processes involving *ANXA2* and *S100A10* (Fig. 8c, d, j). These pathways have been implicated in calcium-dependent membrane dynamics, cytoskeletal remodeling, and vesicular trafficking [43, 44]. The OML also showed elevated expression of plasma-associated proteins (*ALB, HBA*) and the Purkinje-related marker *PCP4L1*, consistent with its anatomical proximity to Purkinje cell dendrites and local vasculature (Supplementary Fig. 6c) [45]. Collectively, these observations point to a compartment defined by increased baseline membrane turnover and synaptic activity. Although these signatures are compatible with a membrane-dynamic compartment near synaptic and perivascular structures, they may also reflect high baseline trafficking and lipid metabolism in active dendritic microenvironments.

SM analysis revealed distinct lipid stratification consistent with the subregional organization of the molecular layer. Although the absolute number of differentially annotated lipid classes varied between sublayers (Fig. 8e), quantitative analysis of the mean abundance of features revealed a specific enrichment of myelin-associated lipids within IML (Fig. 8f). Specifically, phosphatidylethanolamine (PE) and phosphatidylserine (PS) were markedly elevated in IML compared with OML. PE is abundant in myelin membranes and contributes to membrane curvature and packing, while PS, although typically a minor fraction, may influence membrane asymmetry and protein–lipid interactions [43, 46, 47]. It is important to note that the annotation of metabolites based solely on *MALDI-MSI m/z* matching is inherently limited in its ability to resolve isomers or strictly validate chemical identities. Therefore, these findings should be interpreted as reflecting the collective spatial trends of lipid classes, subject to the intrinsic constraints of the annotation resolution of the dataset [3].

In the integrated multiomics landscape, the synergy between the modalities further refined this stratification. Although the molecular-layer subdivision could be suggested from the spatial transcriptomic data alone, ST by itself was insufficient to support a robust, well-resolved, and biologically validated sub-annotation. Compared with ST, in which the IML/OML distinction appeared more diffuse, SP and especially SM showed stronger and more spatially confined layer-specific patterns (Fig. 8j, Supplementary Fig. 6h). Moreover, across different clustering resolutions, SM recovered the IML/OML separation more stably than ST (Supplementary Fig. 6i–k), indicating that the subdivision was less sensitive to clustering parameters in the multimodal setting. Importantly, the metabolomic layer provided orthogonal biological support by highlighting IML-enriched myelin-associated lipid classes, confirming that this subdivision reflects coordinated molecular organization rather than a transcriptomic gradient alone. Although joint ST–SP enrichment analysis successfully identified shared myelin and oligodendrocyte-related pathways in IML, the spatial correlation heatmap revealed distinct modality-specific characteristics: SP exhibited significantly clearer cluster-specific patterning (IML versus OML) compared with ST, which showed more diffuse spatial gradients (Fig. 8g, i). Despite modest spot-level correlations (Supplementary Fig. 6g,h), multiple lipid species showed reproducible, direction-consistent associations with IML-enriched RNA or proteins between spots (Supplementary Fig. 6d–f). The SP–SM correlation analysis identified specific lipid species, including multiple PE molecules, which were strongly tracked with the proteomic signature of IML (Fig. 8h).

In addition, based on the Clustree analysis, we identified two additional stable clustering resolutions at higher granularity, corresponding to cluster numbers of 7 and 12. For each resolution, we visualized the resulting spatial domains together with their corresponding UMAP embeddings. These visualizations suggest that SPOmiAlign has the potential to improve the refinement and delineation of spatial domains (Supplementary Fig. 5b).

Together, these results validate the biological coherence of the identified sublayers and provide an integrated view of tissue organization that covers transcriptional programs, protein expression, and metabolic state.

## Discussion

In this study, we present SPOmiAlign, a modality- and technology-agnostic multimodal spatial alignment framework that enables accurate and scalable registration across heterogeneous spatial multiomic datasets. Building on RoMa, uncertainty-aware correspondence modeling, and coarse-to-fine geometric refinement (Methods), SPOmiAlign overcomes key challenges associated with nonrigid tissue deformation, mismatched spatial resolution, partial tissue overlap between sections, and the absence of shared molecular features. Across a broad range of registration scenarios, including spatial omic-to-CCF registration, spatial multiomic alignment, and tri-omic spatial integration, SPOmiAlign consistently achieved high alignment accuracy while substantially reducing computational cost. Together, downstream biological analyses demonstrate that accurate spatial registration is a fundamental determinant of biological interpretability in spatial multiomic studies.

A central strength of SPOmiAlign lies in its modality-agnostic design, which decouples spatial registration from modality-specific molecular features. By transforming spatial omics data into image-based representations or directly leveraging paired imaging data when available, SPOmiAlign enables a unified alignment strategy across sequencing-based, imaging-based, and mass spectrometry-based spatial technologies. This contrasts with many existing methods that rely on shared gene expression features, manual landmark annotation, or modality-specific assumptions, which often limit generalizability and scalability.

Another key advantage of SPOmiAlign is the use of RoMa for section correspondence. Using a pretrained dense correspondence foundation model, SPOmiAlign infers dense, uncertainty-aware correspondences across entire tissue sections in a single forward pass, rather than relying on iterative optimization over sparse landmarks. This strategy improves robustness to strong nonrigid distortions and weakly textured regions, such as tissue boundaries, providing a reliable basis for precise anatomical annotation and high-quality spatial multiomic integration. Importantly, dense correspondence inference enables anatomically faithful alignment even in regions where molecular signals are sparse or modality-specific, a scenario commonly encountered in real-world spatial omics datasets.

SPOmiAlign is further distinguished by its computational efficiency. Dense correspondence inference followed by lightweight geometric estimation allows alignment to be completed in seconds, avoiding the iterative optimization procedures that characterize many existing alignment tools. Compared with alternative methods, which typically require minutes to hours, SPOmiAlign reduces runtime by two to three orders of magnitude. This efficiency enables scalable applications to high-resolution, atlas-scale datasets and facilitates exploratory or iterative analyses that would otherwise be computationally prohibitive.

Beyond methodological performance, SPOmiAlign provides substantial biological and analytical utility by enabling anatomical standardization of spatial omics data. Automated registration of spatial omics data to a CCF enables standardized anatomical annotation, allowing molecular patterns to be interpreted within a shared anatomical reference across samples, technologies, and studies. This capability is particularly valuable for large-scale atlas projects and cross-dataset comparisons, where manual annotation is impractical and prone to inconsistency. By embedding spatial omics data within a unified anatomical context, SPOmiAlign supports reproducible, region-aware analyses that extend beyond individual experiments.

In addition, accurate spatial registration is a critical prerequisite for integrative spatial multiomic analysis. Our results demonstrate that improved alignment directly enhances multiomic integration, enabling the identification of coherent spatial domains defined by consistent transcriptional, proteomic, and metabolic signatures. In particular, SPOmiAlign-facilitated integration revealed fine-grained spatial domains that are typically obscured by misalignment, underscoring the importance of precise spatial correspondence for uncovering biologically meaningful patterns. However, such interpretations are more reliable at the tissue and regional levels than at extremely fine spatial scales. When different sections contain local distortions, tears, or nonuniform deformations, analyses performed at the level of individual cells or very small spatial regions should be interpreted with caution, as local correspondence may be less precise than the global alignment suggests.

Despite the robust performance of SPOmiAlign, several limitations remain. When paired imaging data are unavailable, SSIs are constructed from discrete spatial spots by rendering each spot with a manually specified radius. Because spatial resolution varies substantially across modalities, the point radius must be adjusted heuristically to reduce discontinuities. Future work could address this limitation by adopting interpolation-based strategies to explicitly fill background regions between discrete spots, such as adaptive kernel density estimation or spatial interpolation. Moreover, the current SSI formulation aggregates high-dimensional molecular features into a single scalar intensity, which prioritizes global structural contrast but may attenuate fine-grained molecular heterogeneity or modality-specific signals. This abstraction reflects an inherent trade-off between geometric robustness and molecular specificity. More broadly, SPOmiAlign is most suitable for applications in which different modalities retain sufficiently informative global morphology or structural correspondence. Under these conditions, the SSI representation and dense correspondence framework can effectively capture anatomically meaningful cross-modal matches even when molecular features are not directly comparable. In future studies, we will focus on designing tissue- or modality-adaptive SSI constructions, including multi-channel or feature-weighted representations, to better capture context-specific structural cues.

To broaden the evaluation beyond brain tissue, we further tested SPOmiAlign on a kidney cancer spatial multi-omics dataset containing paired ST and SM sections. This dataset is more challenging than the mouse brain data because the paired sections exhibit larger deformations, small tears near the tissue boundaries, incomplete tissue regions, and no well-defined anatomical organization. We also examined a cross-technology ST example with partial overlap between MERFISH and Slide-seq hemi-brain sections. These results indicate that SPOmiAlign remains effective on non-brain tissues with irregular morphology, but also highlight that very limited overlap between sections remains a challenging setting and an important current limitation of the method. We also note that SPOmiAlign is more likely to face challenges in tissues with repetitive structures, weak or ambiguous anatomical landmarks, severe distortion, or fragmented tissue regions. In such scenarios, users may benefit from combining SPOmiAlign with additional manual inspection, landmark-based validation, or complementary anatomical priors when available.

Although the current framework benefits from the strong zero-shot generalization ability of pretrained RoMa, domain-specific adaptation could further improve alignment performance for spatial omics data. In our preliminary observations, zero-shot RoMa tends to perform less reliably on particularly challenging slice pairs, especially when overlap is limited to a small central region and substantial peripheral tissue is missing. A rigorous fine-tuning study would require dedicated supervised training data and systematic benchmarking. Nevertheless, domain-specific fine-tuning of dense matching foundation models for sparse and fragmentary spatial omics images is a promising direction for future research.

In summary, SPOmiAlign provides a robust, scalable, and computationally efficient framework for multimodal spatial omics alignment. As spatial multiomics datasets continue to grow in resolution, complexity, and scale, SPOmiAlign offers a practical solution for unifying spatial information across molecular layers and experimental platforms and facilitates a broad range of integrative spatial multiomic studies.

**Key Points**

- We propose SPOmiAlign, the first modality-agnostic spatial multimodal alignment computational framework enabled by a feature matching foundation model.
- Our approach overcomes key limitations of existing methods and achieves accurate alignment under challenging conditions, including partial spatial overlap and nonrigid tissue deformations across multimodal sections.
- Our approach enables automated registration of spatial omics data to anatomical reference atlases, facilitating standardized spatial annotation.
- SPOmiAlign substantially improves downstream integration of spatial transcriptomics, proteomics, and metabolomics data, facilitating spatial domain refinement and the discovery of previously unrecognized functional spatial domains in the mouse brain.
- Our method achieves superior alignment accuracy and spatial multi-omics integration performance compared with existing state-of-the-art spatial alignment methods.

## Supplementary Material

supplementary_bbag331

## Data Availability

All datasets used in this study are publicly available. The Slide-seq dataset, a high-throughput sequencing-based spatial transcriptomics technology with near-cellular resolution comprising 101 adult mouse brain coronal sections that span the entire anteroposterior axis, was obtained from an online resource at https://docs.braincelldata.org/downloads/index.html/Slide-seq_Data. The MERFISH dataset, a high-throughput imaging-based spatial transcriptomics technology with single-cell resolution consisting of 25 adult mouse brain sagittal sections, was obtained from the Allen Brain Cell Atlas at https://alleninstitute.github.io/abc_atlas_access/descriptions/Zhuang-ABCA-3.html. The Allen Common Coordinate Framework (CCF) and the corresponding brain region annotations were obtained from the Allen Brain Atlas. Nissl-stained reference images of the adult mouse brain CCF used for alignment are publicly available for coronal views at https://mouse.brain-map.org/experiment/thumbnails/100048576?image_type=atlas and sagittal views at https://mouse.brain-map.org/experiment/thumbnails/100042147?image_type=atlas. Brain region annotations corresponding to the 25 $\mu$m resolution CCF were downloaded from the file annotation_25.nrrd at https://download.alleninstitute.org/informatics-archive/current-release/mouse_ccf/annotation/ccf_2017/annotation_25.nrrd. The correspondence between the brain region identifiers and the anatomical labels was obtained from structure _tree _safe _2017.csv, available at https://github.com/cortex-lab/allenCCF/blob/master/structure_tree_safe_2017.csv. The MISAR-seq dataset, a spatial multiomic profiling dataset of the mouse brain that jointly measures chromatin accessibility and gene expression, was obtained from a public repository at https://github.com/gpenglab/MISAR-seq/blob/main/Data/Download. The cRCC R114 dataset used in this study, including the spatial transcriptomics and MALDI imaging mass spectrometry-based metabolomics data, was obtained from a public repository at https://zenodo.org/records/14986870. The spatial multiomic mouse brain dataset that integrates MALDI imaging mass spectrometry-based metabolomics, MAGIC-seq spatial transcriptomics, and PLATO spatial proteomics was obtained from a public repository at https://github.com/bioinfo-biols/Flow2Spatial/tree/main/datasets. Source data are provided with this paper. The code for the SPOmiAlign program is available on GitHub at https://github.com/wangyiyuyang/SPOmiAlign. All source code will be released publicly once the manuscript is published.
