# Supplementary material for: SPOmiAlign: a modality-agnostic computational framework for multimodal spatial omics alignment enabled by a feature matching foundation model
Source: Brief Bioinform. 2026 Jun 21;27(3):bbag331. doi: 10.1093/bib/bbag331 (PMC13283438; doi:10.1093/bib/bbag331)
Supplement: supplementary_bbag331 [file supplementary_bbag331.pdf]

Supplementary figures

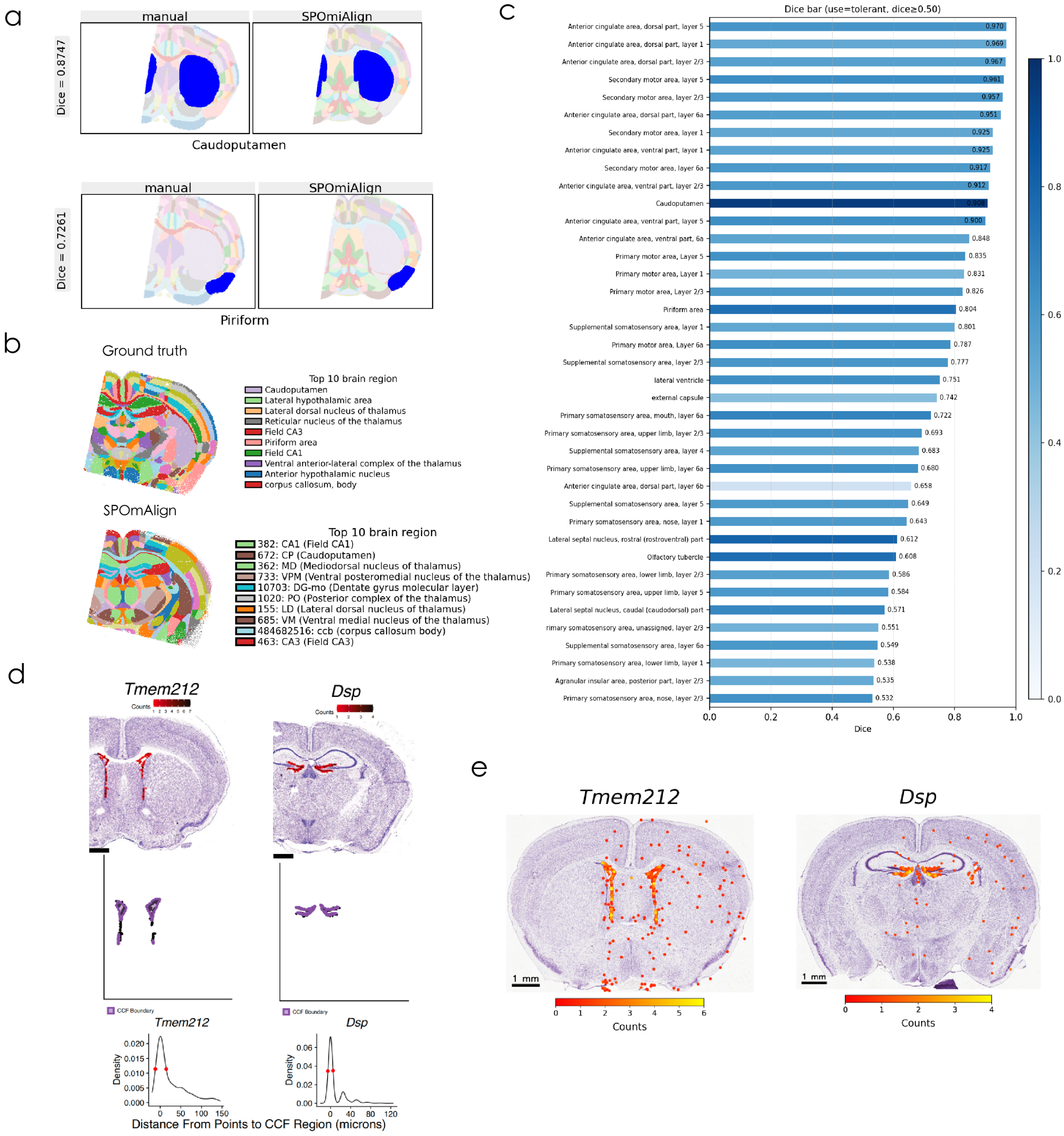

Supplementary Figure 1: Additional evaluation of anatomical annotation accuracy on Slide-seq mouse brain sections. a Dice coefficients for two representative corresponding brain regions (Caudoputamen and Piriform) between SPOmiAlign-derived annotations and ground truth in Slide-seq mouse brain coronal section ID 29. The left panel in each pair shows the ground-truth annotation and the right panel shows the SPOmiAlign-derived annotation. **b** Comparison of anatomical annotations assigned by SPOmiAlign and ground truth across all brain regions in Slide-seq coronal section ID 43, with the top ten regions ranked by area shown in the legends. **c** Dice coefficients for all corresponding brain regions with Dice > 0.5 between SPOmiAlign and ground-truth annotations in Slide-seq coronal section ID 29; bar color indicates normalized region area. **d** Distance density distributions between ground-truth enriched gene-expression spots and the corresponding CCF anatomical boundaries for *Tmem212* and *Dsp*. In the upper spatial maps, red points indicate marker-gene-expressing spots in the spatial transcriptomic slice, and expression levels are encoded by the accompanying colormap. In the lower plots, the purple distributions show the density of distances from expressing spots to the corresponding CCF boundary. **e** Full spatial expression maps of *Tmem212* and *Dsp* across the tissue. Red points indicate expressing spots and the colormap denotes expression level, showing that these genes are specifically enriched near the CCF boundary rather than broadly expressed in surrounding regions.

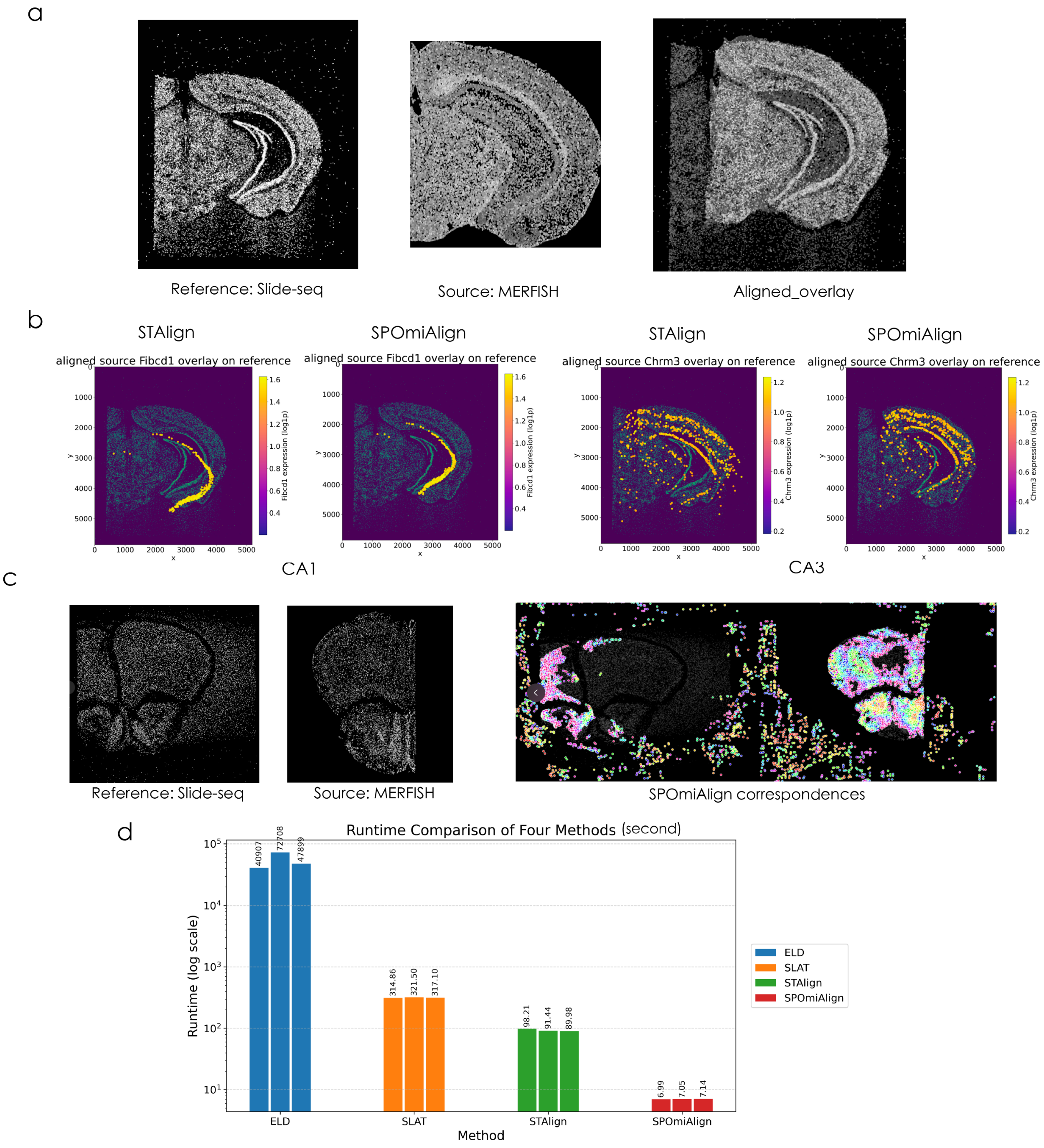

Supplementary Figure 2: Cross-technology spatial transcriptomics alignment, robustness under limited overlap, and runtime comparison. **a** Overlay of the aligned MERFISH slice 092 and Slide-seq slice 57, demonstrating that SPOmiAlign enables cross-technology alignment of brain sections. **b** Spatial distribution of the marker genes *Fibcd1* and *Chrm3* in the aligned sections, showing improved spatial correspondence in the CA1 and CA3 regions after alignment by SPOmiAlign. **c** Alignment of two cross-modality sections with only a small overlapping region, illustrating reduced correspondence accuracy identified by SPOmiAlign in this challenging partial-overlap setting. **d** Runtime comparison of SPOmiAlign, ELD, SLAT, and STAlign on the MERFISH-to-Slide-seq alignment task. Each method was repeated three times on the same high-resolution dataset. Compared with ELD, SLAT, and STAlign, which typically required minutes to hours for alignment, SPOmiAlign reduced computational time by two to three orders of magnitude.

Dataset	Mouse brain spatial two-omic	Mouse brain spatial three-omic	Mouse brain 3D sagittal	Mouse brain 3D cornal	Mouse brain 3D cornal
Modality	Spatial transcriptomic、Spatial ATAC-seq	Spatial transcriptomic、Spatial proteomic、Spatial metabolomic	Spatial transcriptomic	Spatial transcriptomic	Spatial transcriptomic
Technology	MISAR-seq	MAGIC-seq、PLATO、MALDI-MSI	MERFISH	Slide-seq	Slide-seq
Spot number	1939	3908	92497	169683	213885
runtime1(s)	8.4193	9.7285	12.1604	11.3602	11.2004
runtime2(s)	6.1438	9.7763	11.6144	11.4038	11.7882
runtime3(s)	5.0793	9.5984	11.7221	11.6043	11.2525
runtime4(s)	5.2141	6.6163	11.5386	11.5264	11.3965
runtime5(s)	4.4901	8.7012	16.4682	11.2858	11.3575

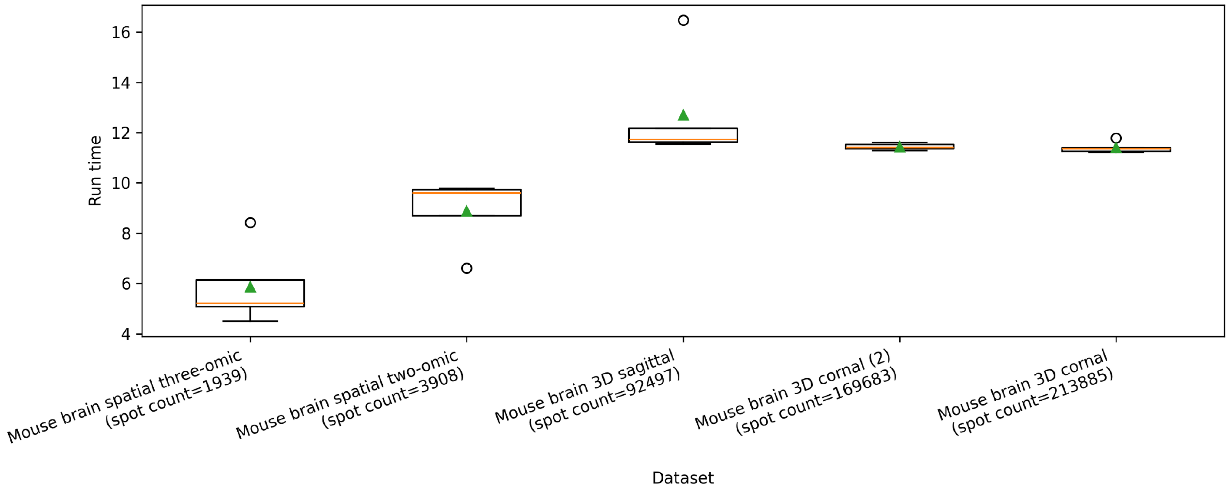

Supplementary Figure 3: Box plots of SPOmiAlign runtime from five repeated runs across five datasets, ordered by increasing spot count. The table above summarizes dataset type, modality, technology, spot number, and the runtime recorded for each repeat. The box plots below show the distribution of runtimes for each dataset, with points indicating individual runs. All experiments were conducted on an Ubuntu 22.04.4 LTS server equipped with one NVIDIA H100 GPU; the onboard ASPEED graphics controller was used only for system display and remote management and did not participate in computation.

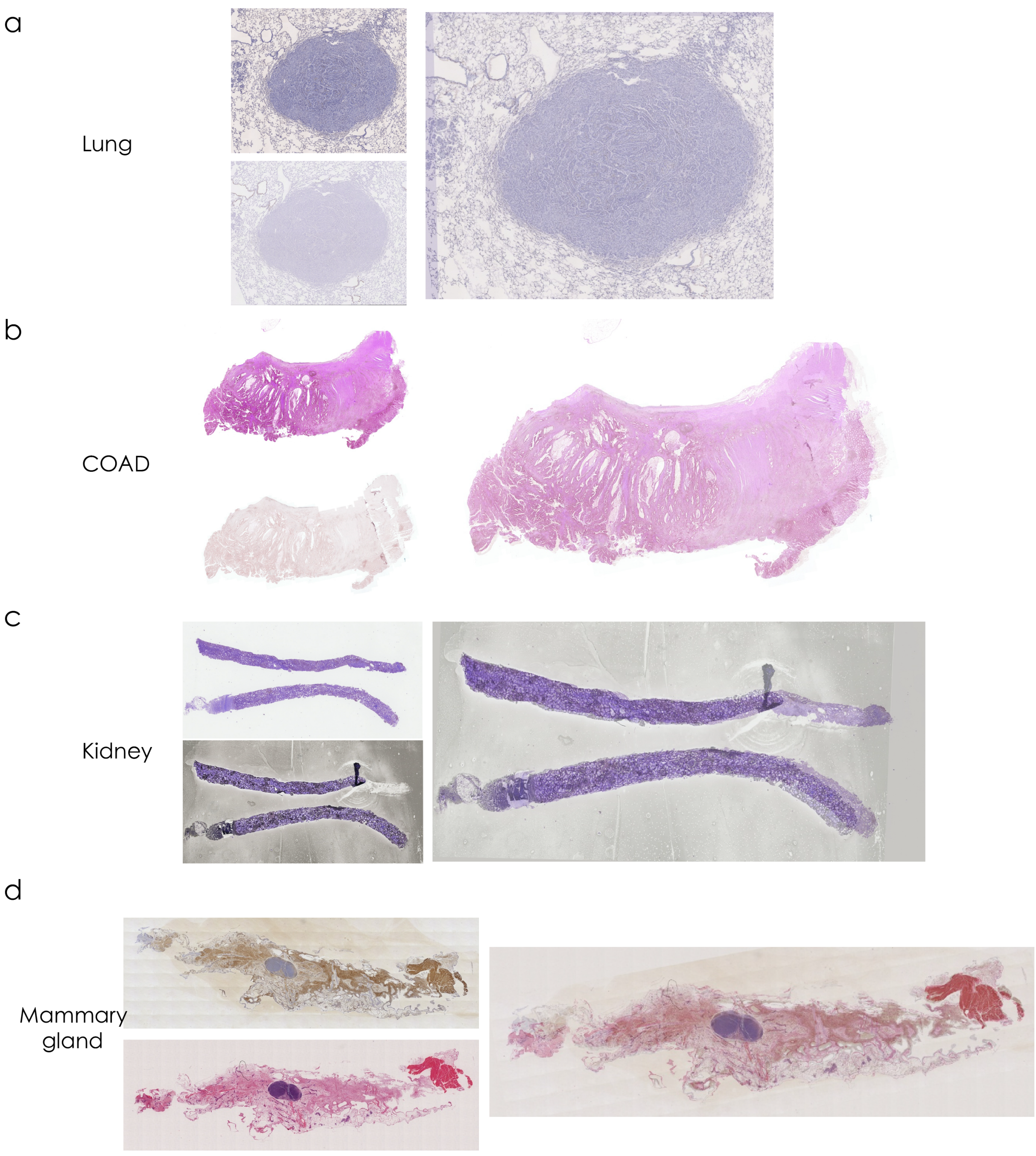

Supplementary Figure 4: Registration of pathological whole-slide images across serial sections from the same tissue in four diseases in ANHIR. a–d Registration of pathological whole-slide images across serial sections from the same tissue in four representative ANHIR cases (lung, COAD, kidney, and mammary gland). In each row, the two panels on the left show the original unaligned whole-slide images, and the panel on the right shows the overlay after SPOmiAlign alignment.

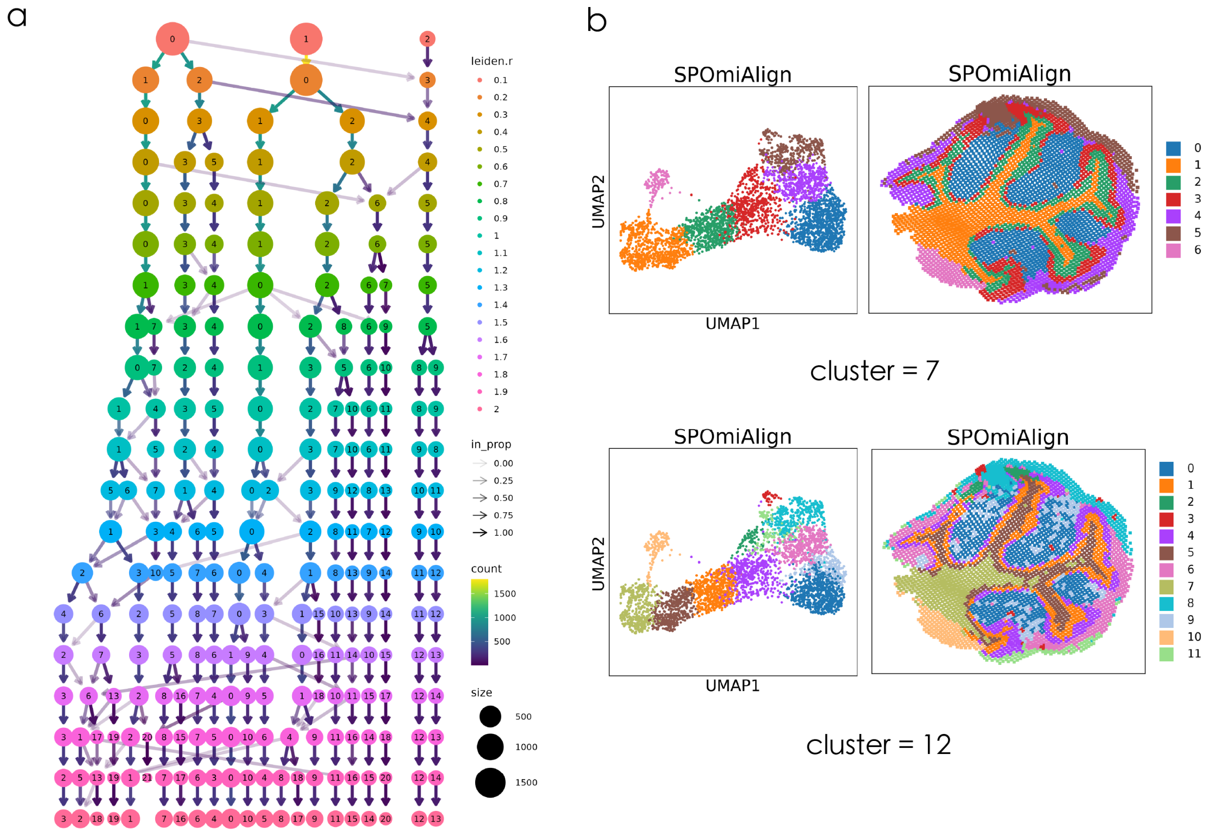

Supplementary Figure 5: Clustree-based assessment of spatial domain stability in integrated tri-omics data. a Clustree representation of clustering hierarchies constructed from the integrated spatial tri-omics dataset across a range of Leiden resolutions. Each node represents a cluster at a given resolution, node size reflects cluster size, and edges connect clusters across adjacent resolutions according to shared membership. **b** UMAP embeddings and corresponding spatial domain maps for two relatively stable clustering configurations (clusters = 7 and 12), illustrating alternative but stable spatial partitioning patterns in the integrated tri-omics data.

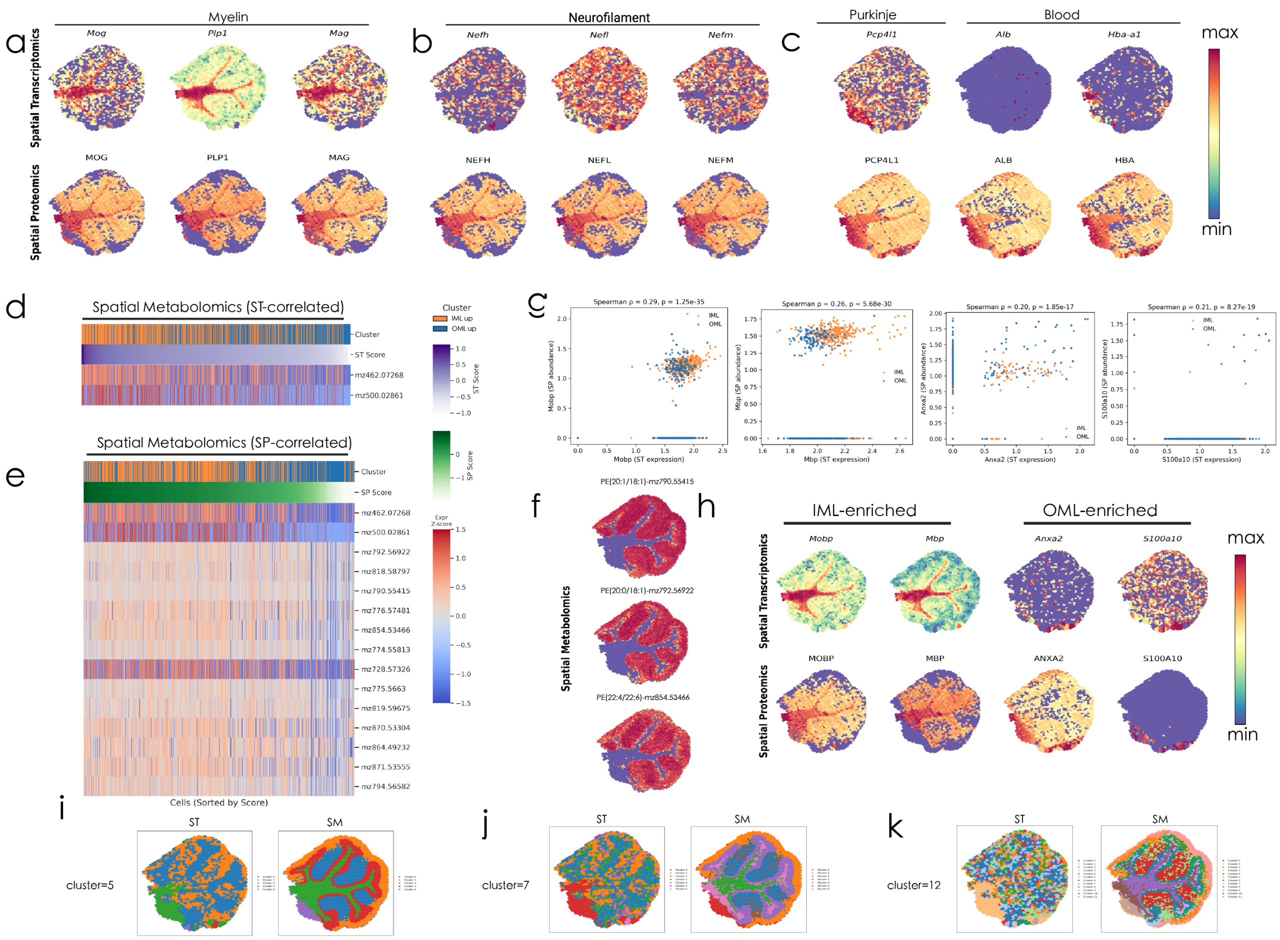

Supplementary Figure 6: Integrated multi-omics characterization of molecular layer substructures. a–c Spatial distribution maps of representative features identified from integrated multi-omics analyses. **a** Myelin- and oligodendrocyte-related genes (*Mog*, *Plp1*, *Mag*) enriched in the inner molecular layer (IML) relative to the outer molecular layer (OML). **b** Neurofilament-related genes (*Nefh*, *Nefl*, *Nefm*) enriched in the IML relative to the OML. **c** Purkinje cell- and blood-related proteins enriched in the OML relative to the IML. **d,e** Heatmaps of non-annotated metabolites identified by correlation analysis with spatial transcriptomics (ST; **d**) and spatial proteomics (SP; **e**) based on SVG–SVP pairs. Columns represent spatial spots ordered by cluster annotation, and rows represent metabolites positively associated with gene-expression or protein-abundance patterns (Spearman correlation > 0.2, FDR < 0.05). **f** Spatial distribution maps of representative PE metabolites identified from the correlated metabolite analysis, showing sublayer-associated spatial patterns. **g** Spearman correlation analysis of representative SVG–SVP pairs for featured genes, illustrating concordance between transcriptomic and proteomic signals. **h** Spatial distribution maps of representative transcripts and proteins identified from integrated multi-omics analyses, demonstrating their concordant localization within molecular layer substructures. Left: IML-enriched features; Right: OML-enriched features. **i–k** Comparison of ST- and SM-derived spatial domains under three clustering configurations (clusters = 5, 7, and 12), showing the consistency and granularity of aligned spatial patterns across modalities.
